# MIF Inhibitor ISO-1 Protects Photoreceptors and Reduces Gliosis in Experimental Retinal Detachment

**DOI:** 10.1038/s41598-017-14298-9

**Published:** 2017-10-30

**Authors:** Bongsu Kim, Rania Kusibati, Tyler Heisler-Taylor, Dimosthenis Mantopoulos, Jiaxi Ding, Mohamed H. Abdel-Rahman, Abhay R. Satoskar, Jonathan P. Godbout, Sanjoy K. Bhattacharya, Colleen M. Cebulla

**Affiliations:** 1 Havener Eye Institute, Department of Ophthalmology and Visual Science, The Ohio State University Wexner Medical Center, Columbus, OH 43212 USA; 2 Division Human Genetics, The Ohio State University Wexner Medical Center, Columbus, OH 43240 USA; 3 Department of Pathology, Menoufia University, Menoufia Governorate, Egypt; 4 Division Experimental Pathology, The Ohio State University Wexner Medical Center, Columbus, OH 43210 USA; 5 Department of Neuroscience, The Ohio State University Wexner Medical Center, Columbus, OH 43210 USA; 6 Bascom Palmer Eye Institute, Department of Ophthalmology, The Miller School of Medicine, Miami, FL 33136 USA

## Abstract

Photoreceptor death and retinal gliosis underlie the majority of vision threatening retinal diseases including retinal detachment (RD). Although the underlying pathobiology of vision limiting processes in RD is not fully understood, inflammation is known to play a critical role. We conducted an iTRAQ proteomic screen of up- and down-regulated proteins in a murine model of RD to identify potential targetable candidates. Macrophage migration inhibitory factor (MIF) was identified and evaluated for neurotoxic and pro-gliotic effects during RD. Systemic administration of the MIF inhibitor ISO-1 significantly blocked photoreceptor apoptosis, outer nuclear layer (ONL) thinning, and retinal gliosis. ISO-1 and MIF knockout (MIFKO) had greater accumulation of Müller glia pERK expression in the detached retina, suggesting that Müller survival pathways might underlie the neuroprotective response. Our data show the feasibility of the MIF-inhibitor ISO-1 to block pathological damage responses in retinal detachment and provide a rationale to explore MIF inhibition as a potential therapeutic option for RD.

## Introduction

Retinal detachment (RD) is a prevalent cause of visual loss, making a major impact on visual ability and quality of life^1^. Diverse retinal diseases may threaten sight because of RD formation including from ocular trauma, retinal tear, retinopathy of prematurity, diabetes, macular degeneration and intraocular tumors^2^. Surgical repair has a high rate of re-attaching the retina; however, permanent visual disability may occur despite prompt and successful surgical repair, with impaired reading vision in 40–60% of patients with macula-affecting RDs^3,4^. Different mechanisms, particularly photoreceptor degeneration and retinal gliosis result in vision loss following detachment. Thus, adjunctive neuroprotective and anti-gliotic strategies to reduce the neurodegeneration induced by RD could potentially benefit multiple diseases.

Given the known importance of inflammation on photoreceptor apoptosis^5,6^, retinal gliosis, and fibrosis^7,8^ after RD, we examined the inflammatory proteins identified in a proteomic screen of a murine model of RD. Macrophage migration inhibitory factor (MIF) was the only cytokine identified. It is pro-inflammatory, playing a key role in cell-mediated immunity^9^ and various autoimmune and central nervous system disorders^10–14^ and its role in RD is unclear. This study reports the results of a proteomic screen of murine experimental RD and evaluates the effects of MIF genetic depletion and pharmacologic inhibition.

## Results

### Proteomic analysis identifies up-regulation of MIF in experimental chronic RD

**Figure 1:**
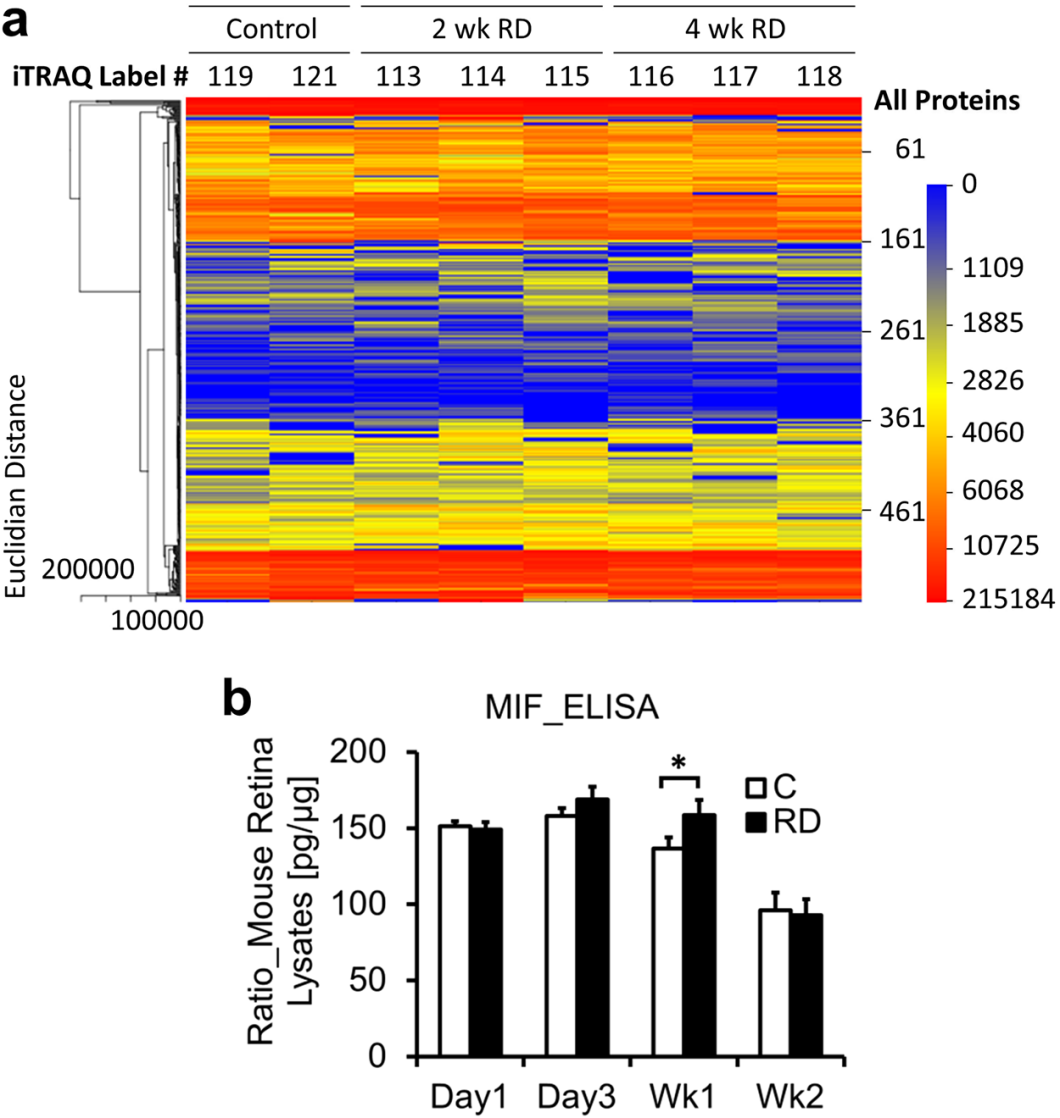
Proteomic analysis of murine RD. (**a**) Heatmap of proteins with FDR < 0.05 identified on iTRAQ proteomic screen. Proteins extracted from individual murine retina plus vitreous at week 2 (n = 3) or week 4 (n = 3) post RD creation were labeled with iTRAQ probes in an 8-plex assay. Control murine retinas (n = 2) were sham-treated (label #119) or untreated (#121). Red represents strong protein expression and blue represents lack of expression. Validation of up-regulated proteins in homogenized retina plus vitreous was performed by ELISA at four timepoints post RD for MIF with n = 15 individual mice/group. MIF expression was significantly elevated in RD eyes at week 1 (**b**, *p = 0.0430).

An iTRAQ proteomics discovery study was performed to evaluate up- and down-regulated proteins in an RD experimental model^15^ at early (2 week, n = 3) and later (4 week, n = 3) timepoints compared to controls (n = 2). 560 proteins with a false discovery rate < 0.05 were identified and relatively quantified (Fig. 1a; Table S1). The heatmap shows subtle differences in protein expression between control, 2 week and 4 week RD conditions.

Due to the known importance of proteins with inflammatory/immunologic function on RD pathology, this subset of proteins (23/560 proteins) was further analyzed (Table 1). No chemokines and only one cytokine were identified on the screen, narrowing the list of targetable, potentially-therapeutic candidates. Many of the strongly up-regulated proteins identified have important cytoprotective and anti-apoptotic functions which could be beneficial in RD. For example, prothymosin alpha, which was up-regulated in 2 week RD retina but not expressed in 4 week RD or controls, is neuroprotective in the retina and brain during periods of oxidative stress and ischemic injury^16,17^, factors which contribute significantly to photoreceptor death in RD.

**Table 1:** Subset of proteins with immune function.

Proteins With Immune Function							
Swiss Protein #	Protein Name	Total Area in Control	Total Area in 2wk Treatment	Total Area in 4wk Treatment	2wk Ratio	4wk Ratio	Percent Coverage
P26350.2	Prothymosin alpha	0	9594.33	0	Undefined	Undefined	20.79
P35700.1	Peroxiredoxin-1	3415.58	12360.24	14324.8	3.62	4.19	46.23
Q60668.2	Heterogeneous nuclear ribonucleoprotein D0	6110.31	18349.77	17466.82	3.00	2.86	45.20
P99026.1	Proteasome subunit, beta type 4	862.11	2389.68	4115.01	2.77	4.77	3.03
Q9D0J8.3	Parathymosin	3814.8	9366.25	17373.49	2.46	4.55	20.38
Q9Z273.1	Tubby like protein-1	4886.93	11038.76	5107.89	2.26	1.05	12.89
Q9R1P4.1	Proteasome subunit alpha type-1	4057.54	8932.56	8932.54	2.20	2.20	27.10
P97797.1	Tyrosine-protein phosphatase non-receptor type substrate 1	35188.13	71413.48	57840.74	2.03	1.64	7.47
P34884.2	Macrophage migration inhibitory factor	9499.3	18097.33	15500.36	1.91	1.63	23.48
Q8R5M8.2	Synaptic cell adhesion molecule	21059.72	38995.5	35280.41	1.85	1.68	28.27
P26645.2	Myristoylated alanine-rich C-kinase substrate	30349.39	55104.61	55970.03	1.82	1.84	3.88
P84086.1	Complexin-2	4181.97	7502.4	4899.99	1.79	1.17	38.81
Q9CZC8.1	Secernin-1	1674.48	2759.81	5907.6	1.65	3.53	16.67
P28798.2	Granulins	3302.48	5032.19	3875.12	1.52	1.17	3.56
P10852.1	4F2 cell-surface antigen heavy chain	2436.96	3693.07	4069.42	1.52	1.67	6.96
P06745.4	Glucose phosphate isomerase-1	23038.51	33157.72	34937.79	1.44	1.52	10.22
P10639.3	Thioredoxin-1	4545.48	6387.62	5411.65	1.41	1.19	10.48
P14206.4	Laminin receptor-1	5603.11	7821.25	4091.14	1.40	0.73	23.73
Q9D0E1.3	Heterogeneous nuclear ribonucleoprotein M	1409.23	1808.8	1457.77	1.28	1.03	4.93
Q11011.2	Aminopeptidase puromycin sensitive	8923.92	11016.61	12161.7	1.23	1.36	1.45
Q9CQM5.1	Thioredoxin domain-containing protein 17	8054.14	9535.02	7485.09	1.18	0.93	8.13
Q9CWS0.3	Dimethylarginine dimethylaminohydrolase 1	16734.91	19661.55	31054.02	1.17	1.86	15.56
Q9JKD3.1	Secretory carrier-associated membrane protein 5	7743.27	7445.48	14437.68	0.96	1.86	6.81

Macrophage migration inhibitory factor (MIF) was the solitary cytokine identified on the screen and was up-regulated in 2 week > 4 week RDs (1.91 and 1.63 fold, respectively). It was attractive for further study since it is associated with worse disease severity for several inflammatory and fibrotic diseases conditions, making MIF inhibitors desirable for clinical development^12,18^. Given the known importance of various inflammatory mediators on photoreceptor apoptosis and scar formation after RD^5,6^ and the negative role of MIF in many diseases with an inflammatory component, the potential role of MIF inhibitors as adjunctive therapy in retinal disease was interesting to explore.

A commercially available inhibitor, ISO-1 is considered a canonical MIF inhibitor in preclinical studies and has beneficially impacted neurologic and inflammatory disease in several animal models^13,19,20^. Thus, subsequent studies evaluated the impact of MIF inhibition in RD including genetic depletion and pharmacologic inhibition.

### Confirmation of MIF iTRAQ result

To confirm that MIF levels were up-regulated in RD, the RD retina + vitreous at day 1, day 3, week 1 and week 2 (n = 15/group) and fellow eye controls was evaluated with ELISA. MIF was expressed at high levels in control retina and increased significantly after RD at week 1 (136.73 ± 7.23 pg MIF/µg total protein in controls vs 158.58 ± 9.87 in RD, p = 0.0430, Fig. 1b).

### Genetic depletion of MIF reduces photoreceptor apoptosis in experimental RD

RDs were created as previously described^15,21^. MIFKO mice with BALB/c background were used. They developed excessive periocular bleeding during the RD procedure (n = 6/10 MIFKO with large hemorrhage and none in background controls). A second study was conducted which used MIFKO mice in a C57BL/6 background and controls (n = 5/group) and the use of cautery to control surface bleeding; none developed hemorrhage.

**Figure 2:**
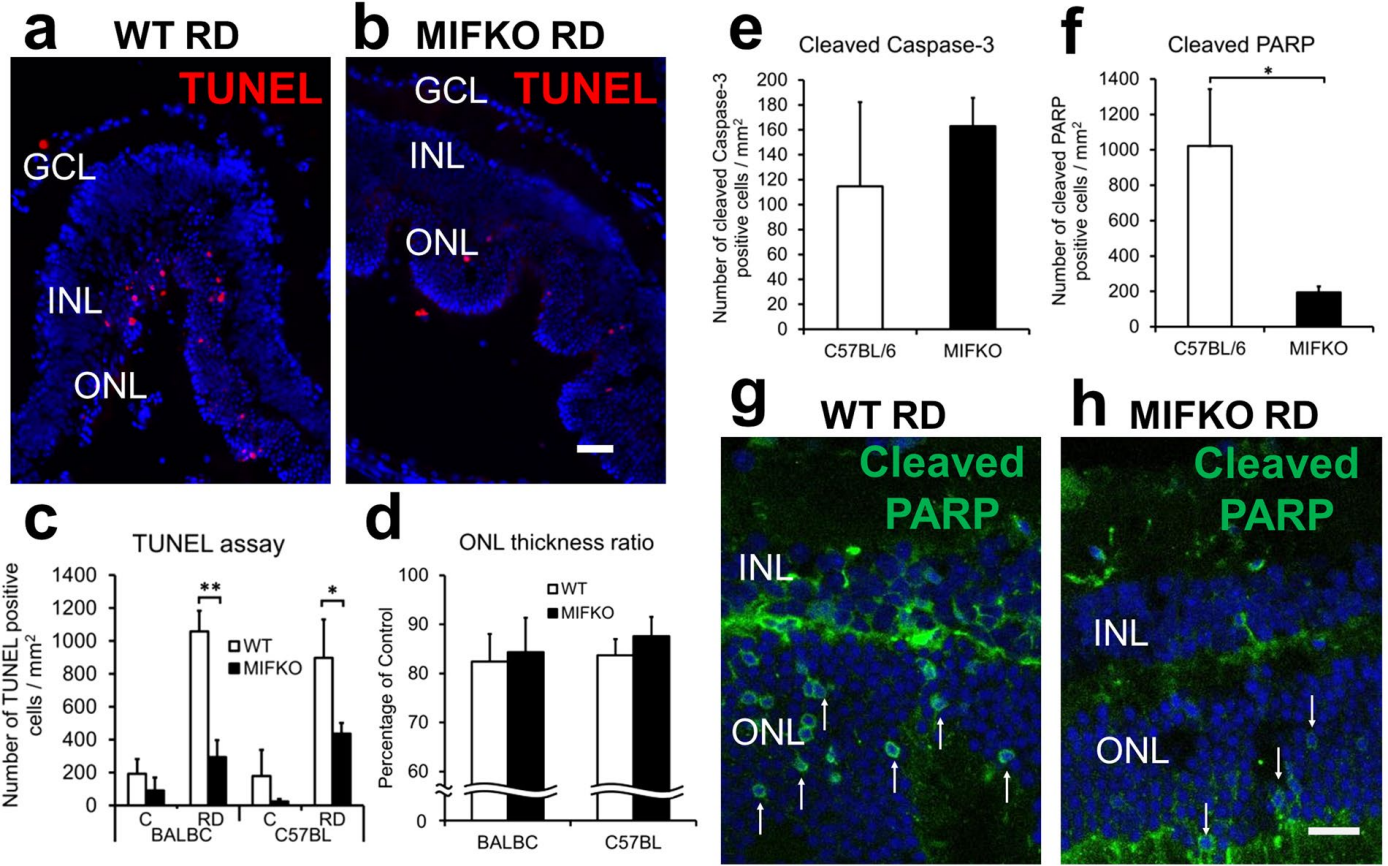
MIF genetic depletion reduces apoptosis but not loss of ONL thickness in RD. MIFKO mice with BALB/c and C57BL/6 backgrounds were used with appropriate background controls (n = 5/group). Representative photographs shows TUNEL-positive cells in day 14 detached retina from BALB/c background controls (**a**) and MIFKO mice (**b**). Scale bar denotes 50 microns. TUNEL-positive cells were significantly reduced in the ONL of day 14 BALB/c and C57BL/6 MIFKO mice (**c**, **p = 0.0008 and *p = 0.0466, respectively). However, the loss of the ONL thickness in these day 14 RDs was not significantly reduced in either BALB/c or C57BL/6 MIFKO mice compared with background controls (**d**, p = 0.4169 and p = 0.2378, respectively). No significant changes were observed in cleaved caspase-3 positive cells (**e**, p = 0.5295). Cleaved PARP-positive cells were significantly reduced in day 14 MIFKO mice (**f**, *p = 0.0224). Confocal micrographs shows cleaved PARP positive cells in day 14 detached retina from C57BL/6 background controls (**g**) and MIFKO mice (**h**). Arrows denote positive cells. Scale bar denotes 25 microns.

The ONL contains the cell bodies of the photoreceptors which undergo cell death after RD and the ONL subsequently becomes thinner as photoreceptors die^22^. To assess the effect of genetic MIF depletion in RD, both TUNEL positive photoreceptors and ONL thickeness measurements were analyzed in both strains of MIFKO mice and their background controls (n = 5/group, Fig. 2a–d). There was a significant reduction in apoptosis by TUNEL assay at day 14 in both the BALB/c mice (1058.40 ± 125.11 cells/mm^2^ background controls vs 293.90 ± 103.22 MIFKO, p = 0.0008) and C57BL/6 mice (897.22 ± 232.97 cells/mm^2^ background controls vs 436.46 ± 65.05 MIFKO, p = 0.0466, Fig. 2c). Surprisingly, this significant reduction in TUNEL accumulation did not translate to a significant protection of the ONL thickness in both BALB/c and C57BL/6 MIFKO mice compared to background controls (p = 0.4169 and 0.2378, respectively, Fig. 2d).

To further explore the impact of genetic MIF depletion on photoreceptor cell death pathways in RD, we analyzed important apoptotic mediators. Activated caspase-3 plays a central role in executing the cleavage of DNA in apoptosis and can be activated by intrinsic and extrinsic apoptosis pathways. We found that the cleaved caspase-3 accumulation in the ONL in detached retina was not significantly different in MIFKO mice vs controls (Fig. 2e). Furthermore, we evaluated the effect of genetic depletion of MIF on accumulation of cleaved poly (ADP-ribose) polymerase (PARP) in detached photoreceptors (Fig. 2f–h). PARP is a multifunctional protein that plays a role in apoptosis, DNA repair, and cell death through caspase-dependent and -independent pathways. Similar to the reduction in TUNEL, MIFKO eyes had significantly reduced cleaved PARP accumulation compared to background controls at day 14 (1021.49 ± 321.37 cells/mm^2^ background control vs 193.59 ± 34.58 MIFKO, p = 0.0224, Fig. 2f). Taken together, these studies on photoreceptor apoptosis and cell death suggest that MIF plays a role in promoting photoreceptor apoptosis during RD, but despite significant reductions in TUNEL and cleaved PARP accumulation, global genetic depletion of MIF could not overcome permanent loss of ONL thickness. To better understand the role of MIF in RD, concurrent studies were undertaken to evaluate the impact of pharmacologic inhibition of MIF.

### Pharmacologic inhibition of MIF protects photoreceptors after experimental RD

**Figure 3:**
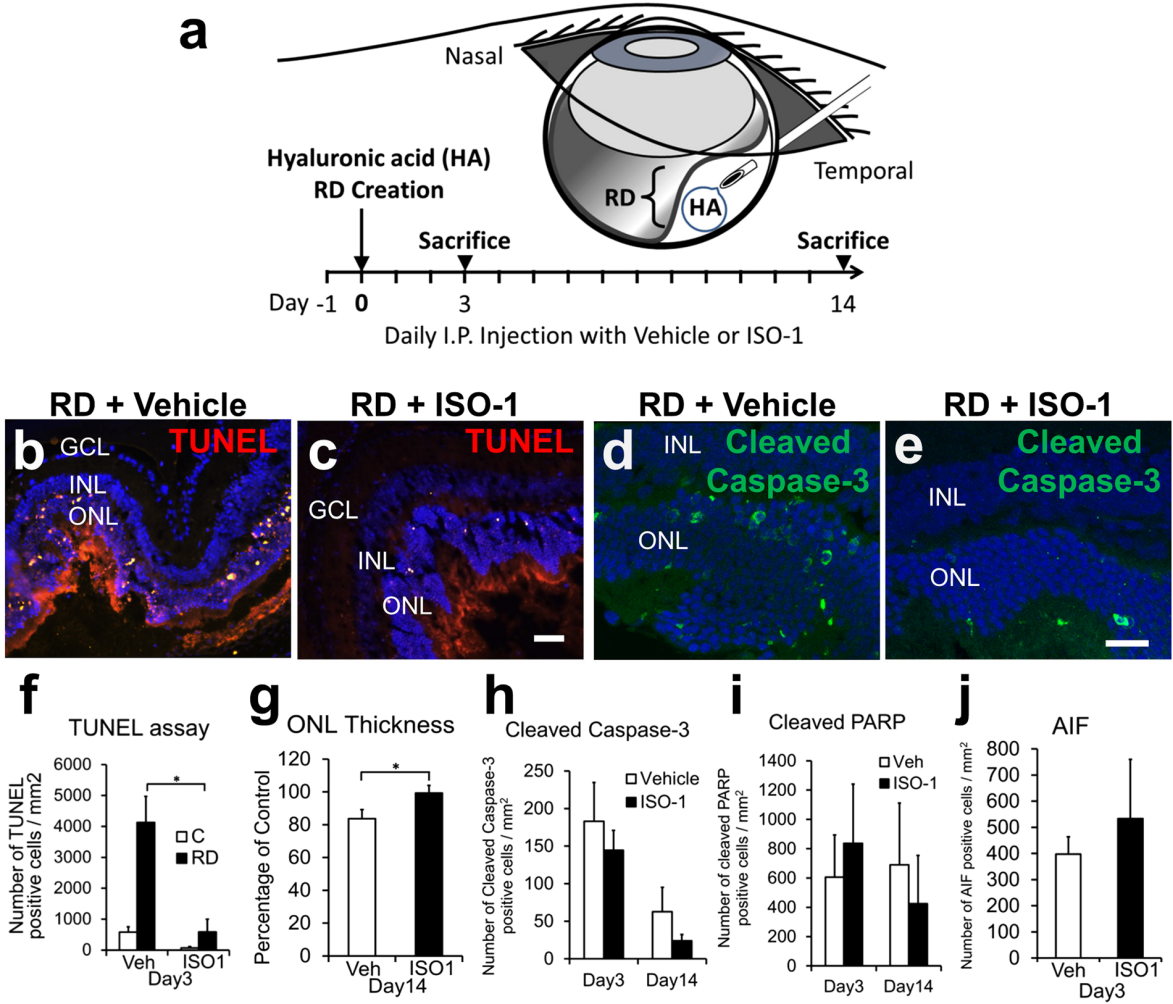
ISO-1 treatment blocks photoreceptor apoptosis and loss of ONL thickness. Experimental design (**a**): ISO-1(40 mg/kg/day) or vehicle treatment was initiated 1 day prior to RD creation and continued daily until sacrifice at day 3 or 14 (n = 6/group). Representative photographs show TUNEL positive cells (red) in the ONL in day 3 detached retina from vehicle (**b**) and ISO-1 treated mice (**c**). Scale bar denotes 50 microns. TUNEL positive cells were significantly reduced in ISO-1 treated mice at day 3 when apoptosis is maximal (**f**, *p = 0.0036), but not different at day 14 (not shown). A second independent study confirmed the significant reduction in TUNEL by ISO-1 (35 mg/kg/day, n = 6/group, p = 0.008, not shown). ONL thickness in ISO-1 treated eyes was significantly thicker than that of vehicle treated eyes at day 14 (**g**, *p = 0.0278). Confocal micrographs shows activated caspase-3 positive cells (green) in the ONL of day 3 background controls (**d**) and ISO-1 treated (**e**) mice (n = 10/group). Scale bar denotes 25 microns. Caspase-3 positive cells were not significantly reduced in ISO-1 treated animals compared to controls in day 3 and 14 (**h**, n = 10/group, p = 0.2593 and p = 0.1367, respectively). Cleaved PARP protein expression in ISO-1 treated eyes was not significantly different from vehicle treated eyes at either timepoints (**i**, n = 6, p = 0.3269 at day3 and n = 5, p = 0.3163 at day14). AIF protein expression in ISO-1 treated eyes was not significantly increased from vehicle treated eyes at day 3 (**j**, n = 6/group, p = 0.2891).

We investigated the ability of systemically administered ISO-1 to prevent apoptosis and photoreceptor death (n = 6, C57BL/6 mice/group, Fig. 3). ISO-1 significantly reduced TUNEL positive cells in the ONL at day 3, when maximal apoptosis is expected (4125.89 ± 849.5 cells/mm^2^ retina vehicle vs 588.68 ± 409.5 ISO-1, p = 0.0036, Fig. 3f). The significant reduction in apoptosis by systemic ISO-1 at day 3 was replicated in an independent experiment using 35 mg/kg instead of 40 mg/kg (n = 6/group, p = 0.008). The higher numbers of TUNEL positive cells in the ISO-1 vs MIFKO studies is best explained by time point; the numbers of TUNEL positive cells are maximal at day 1–3 RDs and decline significantly at later time points (day 14 RD was used in the MIFKO studies to allow evaluation of both TUNEL and ONL thickness in the same sample)^23,24^.

To determine whether ISO-1 could preserve photoreceptors in detached retina over time, the ONL thickness in day 14 RDs was quantitated (n = 6/group). The ONL thickness in the vehicle group dropped significantly to 83.70% ± 5.50 of fellow-eye control while in the ISO-1 group ONL thickness was preserved at 99.28% ± 4.63 (p = 0.0278, Fig. 3g).

### ISO-1 impact on caspase-dependent and -independent apoptosis pathways in RD

To evaluate whether systemic ISO-1 inhibited caspase-3-mediated apoptosis of photoreceptor cells in RD, confocal analysis was performed at day 3 and day 14 (n = 10/group). ISO-1 treatment did not significantly decrease activated caspase-3 positive cells in the ONL, although there was a trend toward a decrease at day 14 (62.68 ± 32.38 cells/mm^2^ retina vehicle vs 23.88 ± 8.51 ISO-1, p = 0.1367, Fig. 3h).

PARP is another component of the cell-death machinery that we hypothesized could be affected by ISO-1. However, confocal analysis of cleaved PARP immunostaining in day 3 and 14 RDs showed no difference in accumulation of cleaved PARP in the ONL in ISO-1 treated eyes compared with controls (606.26 ± 287.78 cells/mm^2^ retina vehicle vs 836.59 ± 404.57 cells/mm^2^ retina ISO-1, p = 0.3269, n = 6 at day3 and 690.53 ± 421.20 cells/mm^2^ retina vehicle vs 424.65 ± 324.84 cells/mm^2^ retina ISO-1, p = 0.3163, n = 5 at day14, Fig. 3i). Caspase-independent mechanisms of apoptosis, such as up-regulation of apoptosis inducing factor (AIF), have been reported to account for a significant portion of the photoreceptor apoptosis in experimental RD^25,26^. To evaluate the potential effect of ISO-1 treatment on AIF expression in RD, we assayed AIF levels on western blot and IHC (n = 6/group). Western immunoblotting quatification showed that ISO-1 did not significantly alter AIF levels compared to vehicle (p = 0.2133, not shown). Similarly, confocal analysis showed AIF accumulation in the ONL was not significantly altered in ISO-1 treated retina compared to vehicle (397.47 ± 66.54 cells/mm^2^ retina vehicle vs 533.18 ± 226.59 ISO-1, p = 0.2891, Fig. 3j).

### ISO-1 does not affect the number of F4/80 + cells after RD

**Figure 4:**
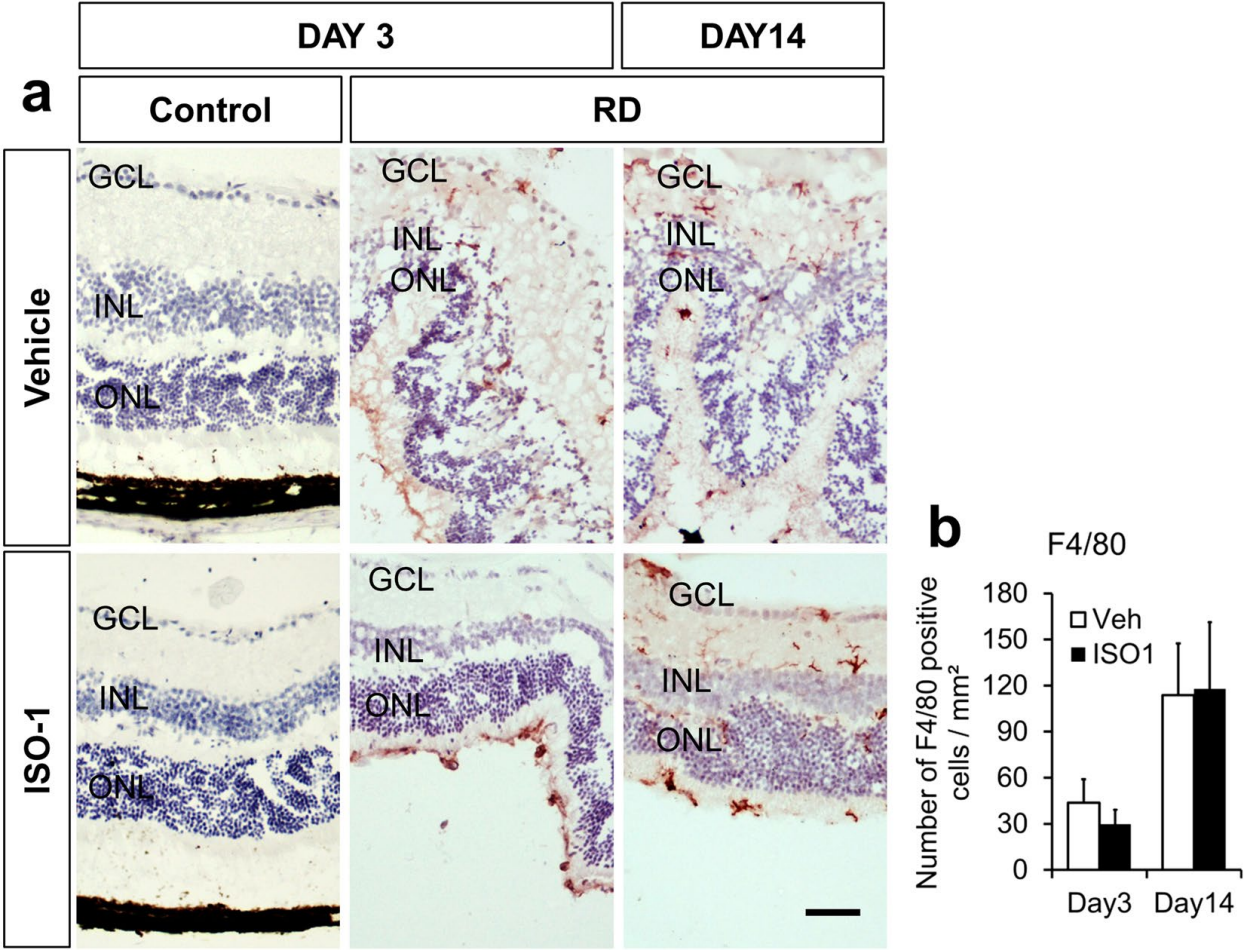
Macrophage accumulation in RD is not affected by ISO-1. (**a**) Immunoperoxidase photomicrographs of F4/80+ cells (red) in the retina of C57BL/6 mice systemically treated with ISO-1 or vehicle demonstrates an accumulation of inflammation in detached retina compared with attached fellow-eye controls at day 3 and 14 (n** = **6/group). (**b**) Quantification reveals the F4/80+ cell count was not affected by ISO-1 treatment compared with vehicle treatment at either timepoints (p = 0.2293, day3 and p = 0.4714, day14). Scale bar denotes 50 microns.

Macrophages and microglia, identified by F4/80 + immunostaining, are recruited after experimental RD^27^. In a liver model, genetic depletion of MIF is associated with decreased F4/80 + cells^28^. Moreover, since macrophages can produce factors, like Monocyte chemoattractant protein-1 (MCP-1), that promote photoreceptor apoptosis in experimental RD^5^, the number of macrophages was quantitated (n = 6 mice/group, Fig. 4a). There was no statistically significant difference in F4/80 + cells between the treatment groups at day 3 (43.73 ± 15.33 cells/mm^2^ retina vehicle vs 29.85 ± 9.45 ISO-1, p = 0.2293) or day 14 (113.80 ± 33.60 cells/mm^2^ retina vehicle vs 117.84 ± 43.42 ISO-1, p = 0.4714, Fig. 4b). Similarly, the macrophage distribution (subretinal space, photoreceptor layer, and ganglion cell layer to ONL) was not significantly different (not shown).

### Expression of GFAP after RD is reduced by ISO-1 and MIFKO

**Figure 5:**
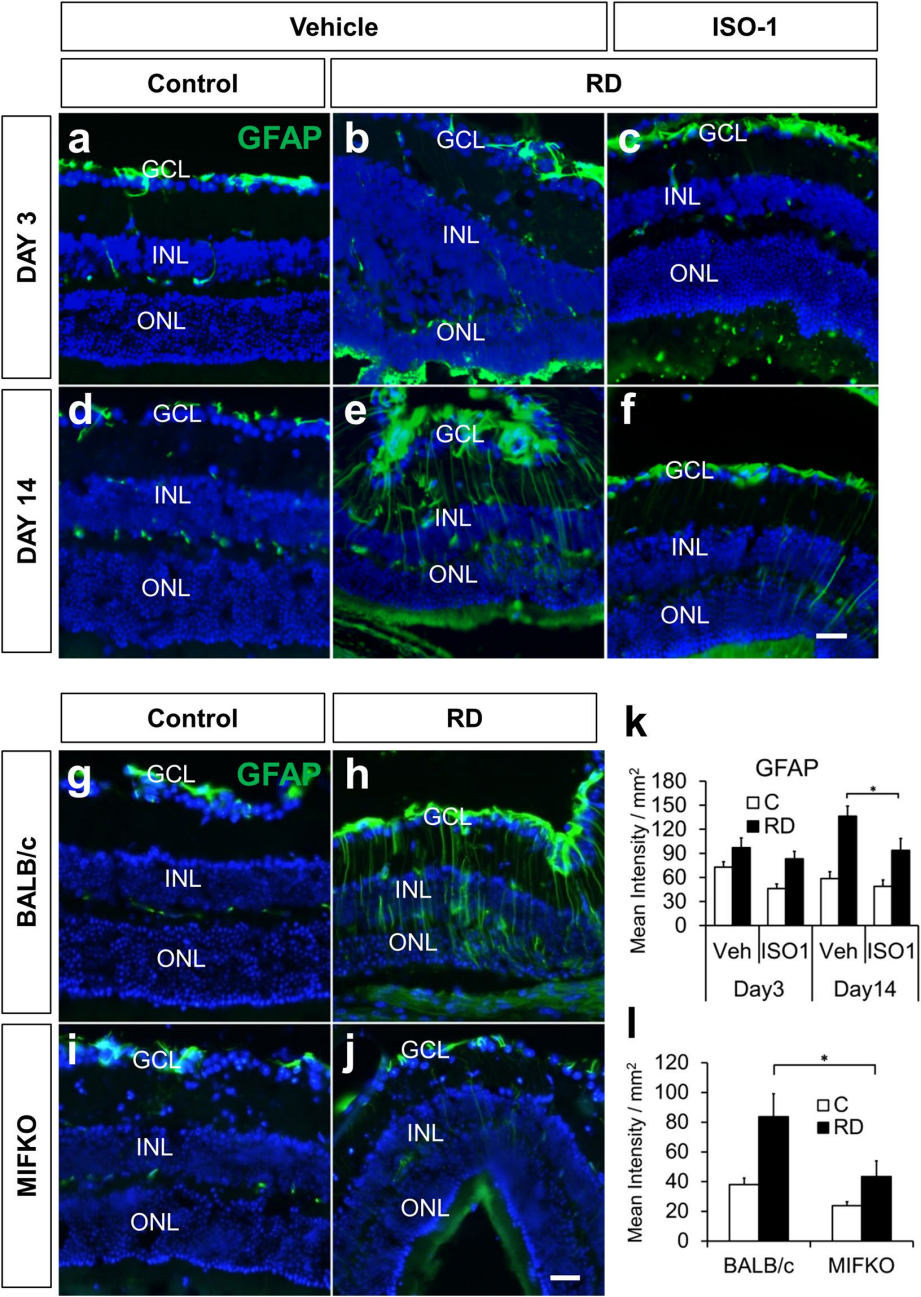
ISO-1 treatment and MIF genetic depletion both reduce retinal gliosis. Immunofluorescence staining shows GFAP accumulation (green) after RD (**b**,**c**,**e**,**f**) compared with attached controls (**a,d**) in vehicle or ISO-1 treated animals at day 3 or day 14 (n = 6/group). Treatment with ISO-1 displayed significant reduction in gliosis in day 14 eyes (**k**, *p = 0.0256) while GFAP expression in day 3 detached retina was not significantly decreased (**k**, p = 0.1898). Detached retinas in day 14 BALB/c wild type controls (**h**) and MIFKO mice (**j**) displayed increased gliosis by GFAP accumulation over adjacent attached retina in controls (**g,i**). Detached retina in MIFKO mice had significantly reduced GFAP accumulation compared with background mice (**l**, n = 5/group, *p = 0.0319). Scale bar denotes 50 microns.

Intermediate filaments like GFAP increase after RD^29–32^ and are a marker for gliotic changes in the retina important to the formation of excessive retinal scarring, a feature of proliferative vitreoretinopathy^33,34^. Moreover, mice with genetic depletion of the intermediate filaments GFAP and vimentin have decreased photoreceptor apoptosis and loss of ONL thickness after RD^32^. To determine the impact of ISO-1 systemic treatment on retinal intermediate filament expression, GFAP immunostaining was performed (n = 6/group, Fig. 5a–f). The normalized GFAP signal intensity was not significantly different in the ISO-1 treated mice at day 3, but was significantly decreased at day 14 (136.40 ± 12.48 mean intensity/mm^2^ retina vehicle vs 93.79 ± 14.69 ISO-1, p = 0.0256, Fig. 5k). Similarly, MIFKO retinas (n = 5/group) had significantly less GFAP reactivity at day 14 than background controls (83.69 ± 15.47 mean intensity/mm^2^ retina BALB/c control vs 43.44 ± 10.54 MIFKO, p = 0.0319, Fig. 5l).

### Pharmacologic inhibition of MIF increases retinal pERK accumulation after RD

**Figure 6:**
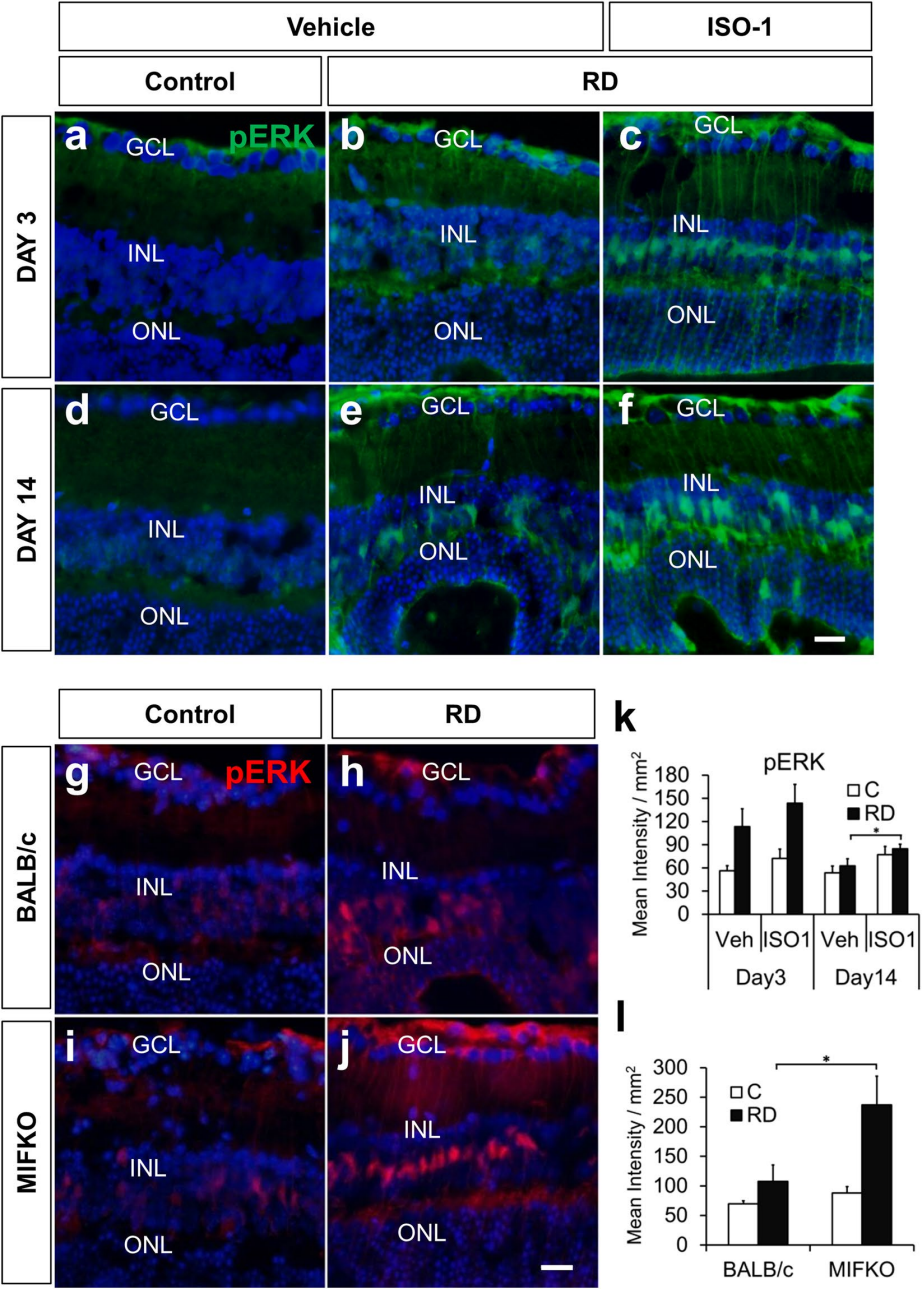
Retinal pERK is up-regulated in ISO-1 and MIFKO RDs. Immunofluorescence staining shows pERK (green) accumulation in Müller glia after RD (**b,c,e,f**) compared with attached controls (**a,d**) in vehicle or ISO-1 treated animals at day 3 or day 14 (n = 6**/**group). Treatment with ISO-1 displayed significant elevation in pERK expression in day 14 eyes (**k**, *p = 0.0356) while pERK expression in day 3 detached retinas was not significantly elevated (**k**, p = 0.1948). Detached retinas in day 14 BALB/c wild type controls (**h**) and MIFKO mice (**j**) displayed increased pERK accumulation (red) compared to attached retina in controls (**g,i**). Detached retina in MIFKO mice had significantly elevated pERK accumulation compared with background mice (**l**, n = 5/group, *p = 0.0246). Scale bar denotes 50 microns.

Levels of phosphorylated ERK1/2 increase in retinal Müller cells and astrocytes after experimental RD^32^. Several MIF functions have been linked to intracellular signaling by ERK1/2 phosphorylation^35,36^ and it is unknown if ISO-1 could have any effect on retinal pERK accumulation. We hypothesized that pERK levels would be diminished by MIF inhibition during RD. To test this, we evaluated retinal pERK levels in vehicle- and ISO-1 treated murine RDs (n = 6/group, Fig. 6a–f). Immunofluorescence analysis showed that ISO-1 treatment was actually associated with an increase in retinal pERK levels in Müller glia compared with vehicle-treated RD retinas at day 14 (62.55 ± 9.16 mean intensity/mm^2^ retina vehicle vs 84.62 ± 5.97 ISO-1, p = 0.0356, Fig. 6k). The identity of Müller-glia progenitor cells was confirmed with Sox-2 immunofluorescence (not shown). Similarly, MIFKO mice at day 14 (n = 5/group) showed increased retinal pERK accumulation in RD (107.68 ± 27.60 mean intensity/mm^2^ retina BALB/c control vs 237.01 ± 48.54 MIFKO, p = 0.0246, Fig. 6l). Thus, ISO-1 treatment and MIF genetic depletion surprisingly had enhanced accumulation of pERK in the Müller glia during RD. Interestingly, several studies show that accumulation of retinal ERK1/2 phosphorylation in different models of retinal damage can be neuroprotective for photoreceptors^37,38^ and it is tempting to speculate that up-regulation of Müller survival pathways could be a mechanism underlying ISO-1 neuroprotection during RD.

### ISO-1 does not significantly alter macrophage polarization in RD

Inacio *et al*. showed that ISO-1 was neuroprotective in a stroke model in rats, which correlated with a change in the quality rather than quantity of macrophages/microglia in the infarcted brain^13^. Macrophages and microglia have functional characterizations known as M1 polarization (producing pro-inflammatory products such as inducible nitric oxide synthase (iNOS)) or M2 polarization (characterized by a wound-healing function with products such as arginase-1 (Arg1))^39^. We investigated the expression of inflammatory mediators and M1 vs M2 gene expression in the retina + vitreous of vehicle or ISO-1-treated RD mice at day 3 post detachment (n = 9–11 mice/group, Fig. S1a **)**. We found no significant difference in M1 or M2 gene expression induced by systemic ISO-1 treatment in this RD model. Similarly, immunostaining for Arg1 in macrophage hot-spots in detached retina revealed no significant difference in the proportion of Arg1 positive macrophages between the groups (n = 12–13mice/group, Fig. S1b **)**.

## Discussion

Proteomic iTRAQ screening identified several differentially expressed proteins in RD retina relevant to neuroprotection. Herein we focused on the proinflammatory cytokine MIF for its translational value; other interesting candidates will be explored in future studies.

MIF had a strong rationale for further exploration as a potential therapeutic target in RD. Its expression is associated with worse disease severity for several inflammatory and fibrotic diseases^12,18^. We demonstrated findings relevant to possible clinical translation: that systemic administration of the MIF-inhibitor ISO-1 blocked photoreceptor apoptosis and ONL thinning in detached retina. These results suggest a neuroprotective effect of the drug and identify MIF as a targetable molecule to potentially impact visual loss from RD.

Matsuda *et al*. has also shown that MIF may play a role in the regulation of ocular immunity and inflammation. In rat, they showed that MIF is expressed in the nonpigmented ciliary and iris epithelium^40^. In the rat cornea, MIF is expressed in the basal epithelium and endothelium and it is rapidly upregulated in the cornea and aqueous humor after penetrating injury^41^. MIF was also identified in human corneal epithelium and endothelium, suggesting a potential role in corneal immunity and uveitis^42^. In fact elevated MIF levels were detected in serum of patients with uveitis, with highest levels during active phases^43,44^. Posteriorly, Matsuda *et al*. identified constitutive MIF expression in the rat retina, primarily in the astrocytes, Müller glia, and RPE^45^.

In accordance with our findings of MIF in experimental RD, similar effects of MIF inhibition have been shown in other CNS disease models, including experimental stroke^13^, experimental autoimmune encephalitis^46^, and NMDA-induced retinal damage^47^ but not in a model of optic nerve injury^48^ or some other models of stroke^49,50^. Notably, the present study utilized female mice for the ISO-1 experiments and found a neuro-protective effect. In contrast, the study by Turtzo *et al*. found that MIF genetic depletion was deleterious in female mice compared to males after stroke. These findings suggest that there are likely different tissue- and model-specific neuroprotective effects of MIF as well as possible sex differences. Future studies should further evaluate these biologically relevant variables on MIF retinal biology.

Inflammation is one of the main mechanisms that lead to photoreceptor death after RD^5^ and MIF may contribute to the inflammatory cascade by inhibiting apoptosis in monocytes/macrophages^51^. Moreover, MIF inhibition or genetic depletion blocks the accumulation of infiltrating macrophages in some disease models^20^. However, in our model, the accumulation of F4/80 + macrophages located in the detached retina was not significantly altered by ISO-1. A possible explanation is that since MIF exists pre-formed in monocytes/macrophages^52^ and its pro-inflammatory action cannot be sufficiently prevented, at least not in the early stages after the detachment. Other studies of MIF in the CNS showed that MIF depletion was neuroprotective in a cerebral infarct model without changing the quantity of accumulating macrophages but by altering their surface expression^13^. Although we predicted that the macrophage phenotype would be shifted to a more M2-predominant polarization by ISO-1, levels of select M1 and M2 retinal gene expression were not significantly altered by ISO-1 treatment, nor was the fraction of arginase-1 positive M2 macrophages in the detached retina. These results do not exclude the possibility that inflammatory effects of MIF play a role in inducing neurodegeneration in RD and perhaps a different time point would allow detection of an effect. Future work will further evaluate the impact of timing and microglia depletion on MIF inhibitor-mediated neuroprotection.

The mechanism of ISO-1 neuroprotection during RD is unknown. Interestingly we found that in the MIFKO experiments, TUNEL and cleaved PARP were significantly reduced in the knockout mice, while cleaved caspase-3 did not reach significance. In the ISO-1 studies, we found that TUNEL was significantly reduced and ONL thickness was significantly protected by ISO-1, while caspase-3, cleaved PARP, and AIF (a caspase-independent mediator of apoptosis) were not significantly different between groups. These findings show that MIF inhibition in experimental RD has neuroprotective effects, yet the exact mechanism is unclear and may be different between the pharmacologic and global MIF depletion conditions. In the ISO-1 treated mice, there is not total depletion of MIF, compared to the MIF knockout. The ISO-1 treated mice are likely more physiologically relevant to what we would expect in the human. We are continuing work to better evaluate the relationship of MIF with caspase-dependent and independent cell death pathways in the retina.

Elegant studies by White *et al*.^53^ may contribute a potential explanation for variable cytoprotective results in MIF depletion studies due to the importance of the “compartmentalization” of MIF. In their murine myocardial infarction model they explored the importance of MIF expressed by the stromal cell compartment of the damaged tissue (cardiac myocytes) versus the bone-marrow-derived cell compartment. Chimeric MIF knockouts (MIFKO) were created so that the resident cells of the body remained either MIF wild-type (WT) or KO, and received either MIF WT or KO bone-marrow-derived cells. After myocardial infarction, having MIF WT cardiac myocytes and MIFKO bone marrow was cardioprotective, even more than global MIFKO in both compartments. In contrast, mice with MIF WT bone marrow and MIF KO cardiac myocytes had an increased risk of cardiac rupture after myocardial infarction even more than wild type mice. Liehn *et al*. showed similar results with CXCR2 chimeras^54^. It is possible that different compartments of MIF expression could be important in the retina as well. Interestingly, there is high baseline retinal MIF expression, particularly in the astrocytes and Müller glia^45^ and the functional role of MIF in this compartment is unknown. It is possible that Müller MIF might have a similar cytoprotective role as seen in the cardiac myocytes and may help explain why global genetic depletion of MIF did not produce as robust neuroprotection as the MIF inhibitor ISO-1. Future studies with Müller-selective MIF depletion as well as depletion of microglia/macrophages should investigate this, particularly as MIF inhibition may be considered therapeutically.

Another candidate neuroprotective mechanism of MIF inhibition was the up-regulation of the MAPK Müller survival pathway, which is neuroprotective for photoreceptors in different damage models^37,38,55,56^. Both ISO-1 and MIF genetic depletion enhanced the accumulation of pERK in the Müller glia during RD, which may promote photoreceptor survival. Future studies must be conducted to further elucidate the underlying mechanism of protection.

Proliferative vitreoretinopathy (PVR) is a feared complication after RD with excessive gliosis, scar formation, and severe vision loss. The activation of Müller glia and up-regulation of intermediate filaments like GFAP are particularly significant in PVR formation as well as in RD, with the intensity of staining correlating with the severity of detachment^34^. In our experiments, treatment with ISO-1 and genetic depletion of MIF both significantly decreased GFAP signal intensity, suggesting that retinal gliosis could be mitigated by MIF inhibition. This result is similar to findings in a neurodegenerative Alzheimer’s murine model in which MIF genetic depletion or blockade by ISO-1 decreased CNS GFAP levels^57^. In contrast, GFAP levels were not decreased in MIF knockouts in an experimental stroke model and the authors concluded that MIF is part of the gliotic process but not essential for it^58^.

Our preclinical ISO-1 data have clinical relevance to PVR; MIF levels are significantly elevated in the vitreous and subretinal fluid of patients with RD developing PVR compared with those without PVR^59,60^. Thus, there is a rationale for future clinical trials with MIF inhibitors, particularly for high-risk detachments.

Other prevalent vision-threatening retinal diseases such as age-related macular degeneration and diabetic retinopathy could also benefit from inhibition of gliosis as well as photoreceptor apoptosis and such strategies are being considered in the development of novel therapeutics for these diseases^61,62^. Proliferative diabetic retinopathy in particular may benefit from MIF inhibition; ocular MIF levels are elevated in patients with diabetic retinopathy, with the highest level in proliferative disease with the greatest degree of fibrosis, suggesting that MIF may play a role in the fibrovascular proliferative process^63,64^.

Because surgical repair of RD alone is inadequate to prevent loss of photoreceprtors, additional treatment options aimed at the inflammatory and gliotic component of the neurodegenerative process may be valuable to reduce vision loss. To the best of our knowledge, this is the first study where ISO-1 was used as an inhibitor of MIF in an experimental model of RD. Our findings suggest a rationale to develop and trial MIF inhibitors as adjunctive therapeutics for patients with retinal detachment and other retinal diseases.

## Methods

### Reagents

ISO-1 ((S, R)-3-(4-hydroxyphenyl)-4, 5-dihydro-5-isoxazole acetic acid methyl ester) was obtained from EMD Millipore (Billerica, MA) and diluted in 10% DMSO for *in vivo* studies.

### Animals and Retinal Detachment Surgery

This research adheres to the principles of the ARVO Statement for the Use of Animals in Ophthalmic and Vision Research. It was conducted under a protocol approved by The Ohio States University Institutional Animal Care and Use Committee. Similar to our previous studies, adult female C57BL/6 mice (age 16–31weeks) were purchased from The Jackson Laboratory (Bar Harbor, ME)^15^. Better quality RDs are generated in mice aged 16 weeks or older (unpublished observation). For genetic depletion studies, BALB/c and C57BL/6 control mice and previously described *Mif* ^-^ knockout (MIFKO) mutants^65^ backcrossed > 10 generations to a BALB/c and C57BL/6 background (age 16–20 weeks) were used.

Retinal detachments (RD) were induced in anesthetized mice by subretinal injection of undiluted hyaluronic acid (HA, 10 mg/ml, Abbott Medical Optics, Abbott Park, IL) into left eyes as previously described^21^.

### ISO-1 Studies

Starting 24 hours prior to RD induction, C57BL/6 mice were treated with daily intraperitoneal injections of freshly prepared ISO-1, (40 mg/kg diluted in saline with 10% DMSO) or vehicle until the day of euthanasia, similar to the protocol of Leng *et al*.^20^. A second study at 35 mg/kg was performed with similar results.

### Enucleation, Fixation, and mRNA and Protein Isolation

Anesthetized mice were euthanized and eyes enucleated. Retinas, with vitreous, were dissected under an operating microscope using 0.12 forceps and Vannas scissors to cut the sclera and retina posterior to the limbus and remove the anterior cap of the eye. In the remaining posterior globe, the retina was gently separated from the underlying RPE and choroid and was cut near the insertion into the optic nerve. Retinas that were processed for RNA were dissected with instruments wiped with RNase-Zap and retinas were placed in TRIzol. For protein isolation retinas were placed in ice-cold 1x cell lysis buffer (#9803 S, Cell Signaling) freshly prepared with protease inhibitor cocktail set (#539134, Calbiochem) and 1 mM of phenylmethylsulfonyl fluoride (#AC215740010, Acros Organics)^15^.

### iTRAQ labeling

Protein lysates from each retina were quantitated as previously described^15^. The retina + vitreous protein extracts (100 µg of protein per sample) were diluted with 50 mM TEAB, incubated with 2% SDS denaturant and 5 mM TCEP reducing agent. Fresh iodoacetamide (84 mM) was added and incubated before the addition of sequencing grade trypsin (Promega) for overnight incubation at 37 °C. The digests were dried in a vacuum centrifuge and iTRAQ labeling was carried out according to the instructions in the iTRAQ labeling kit (iTRAQ 8-plex One Assay Kit, Penn State University Proteomic Core).

### 2D –LC and Mass spectrometry

Liquid chromatography (LC) 2D separation and tandem mass spectrometry was performed at Penn State University Proteomic Core Facility. 2D-LC separations: SCX Separations were performed on a passivated Waters 600E HPLC system, using a 4.6 × 250 mm Polysulfoethyl aspartamide column (PolyLC, Columbia, MD) at a flow rate of 1 ml/min. Buffer A contained 10 mM ammonium formate, pH 2.7, in 20% acetonitrile/80% water. Buffer B contained 666 mM ammonium formate, pH 2.7, in 20% acetonitrile/80% water. The gradient was Buffer A at 100% (0–22 minutes following sample injection), 0% → 40% Buffer B (16–48 min), 40% → 100% Buffer B (48–49 min), then isocratic 100% Buffer B (49–56 min), then at 56 min switched back to 100% A to re-equilibrate for the next injection. The first 26 ml of eluant (containing all flow-through fractions) was combined into one fraction, then 14 additional 2-ml fractions were collected. All 15 of these SCX fractions were dried down completely to reduce volume and to remove the volatile ammonium formate salts, then resuspended in 9 µl of 2% (v/v) acetonitrile, 0.1% (v/v) trifluoroacetic acid and filtered prior to reverse phase C18 nanoflow-LC separation. For 2nd dimension separation by reverse phase nanoflow LC, each SCX fraction was autoinjected onto a Chromolith CapRod column (150 × 0.1 mm, Merck) using a 5 µl injector loop on a Tempo LC MALDI Spotting system (ABI-MDS/Sciex). Buffer C was 2% acetonitrile, 0.1% trifluoroacetic acid, and Buffer D was 98% acetonitrile, 0.1% trifluoroacetic acid.

The elution gradient was 95% C/ 5% D (2 µl per minute flowrate from 0–3 min, then 2.5 µl per minute from 3–8.1 min), 5% D → 38% D (8.1–40 min), 38% D → 80% D (41–44 min), 80% D → 5% D (44–49 min) (initial conditions). Flow rate was 2.5 µl/min during the gradient, and an equal flow of MALDI matrix solution was added post-column (7 mg/ml recrystallized CHCA (a-cyano-hydroxycinnamic acid), 2 mg/ml ammonium phosphate, 0.1% trifluoroacetic acid, 80% acetonitrile). (Gradient used prior to 2008 was 100% C (0–4 min), 0 → 10% D (4–10 min), 10% → 25% D (10–30 min), 25% → 40% D (30–35 min), 40% → 80% D (35–38 min), 80% D (38–42 min), 80% → 0% D (42–43 min), 0% D (43–50 min), with a flow rate of 3.0 µl per minute with an equal flow of MALDI matrix solution.) The combined eluant was automatically spotted onto a stainless steel MALDI target plate every 6 seconds (0.6 µl per spot), for a total of 370 spots per original SCX fraction.

### Mass Spectrometry

After sample spot drying, thirteen calibrant spots (ABI 4700 Mix) were added to each plate manually. MALDI target plates (15 per experiment) were analyzed in a data-dependent manner on an ABI 5800 MALDI TOF-TOF. As each plate was entered into the instrument, a plate calibration/ MS Default calibration update was performed, and then the MS/MS default calibration was updated. MS Spectra were taken from 5500 MALDI Spots, averaging 500 laser shots per spot at laser power 2600. A plate-wide interpretation was then automatically performed, choosing the highest peak of each observed m/z value for subsequent MS/MS analysis. A total of 8118 MS/MS spectra were taken with up to 2500 laser shots per spectrum at Laser Power 3100, with CID gas Air at 1.2 to 1.3 × 10–6 Torr.

Up to 2500 laser shots at laser power 3100 were accumulated for each MS/MS spectrum. After the MS and MS/MS spectra from all 15 plates in a sample set had been acquired, protein identification and quantitation were performed using the Paragon algorithm as implemented in Protein Pilot 3.0 software (ABI/MDS-Sciex). Protein Pilot Software was utilized for searches with the following search parameters: Cys Alkylation – Iodoacetamide; ID Focus – Biological Modifications; Search Effort – Thorough. The database searched was Jan 1st 2010 mouse NCBI database Sequences containing 256336 Protein Sequences, plus 156 common lab contaminants. For estimation of false discovery rate (FDR), a simultaneous search was performed of a concatenated Decoy database which is the exact reverse of each protein sequence in the database plus 156 common human and lab contaminants (ABSciex_ContaminantDB_20070711). Total protein sequences searched in the database plus contaminants plus concatenated reverse decoy database was 512,156. The protein identifications at 95% confidence level were retained. The preset “Thorough” (iTRAQ or Identification) search settings were used where identifications must have a ProteinPilot Unused Score > 1.3 ( >95% Confidence interval). In addition, the only protein IDs accepted had a local “FDR” estimation of no higher than 5%, as calculated from the slope of the accumulated Decoy database hits by the PSPEP (Proteomics System Performance Evaluation Pipeline) program^66^.

One-matrix heatmaps of the data were generated using CIM Miner (http://discover.nci.nih.gov/cimminer/) by plotting the area of each protein with FDR < 5% for each iTRAQ group. Clustering was performed using the Euclidean distance method with the Average Linkage algorithm. The column order was kept fixed for the analysis. Fold-change was determined by calculating the ratio of the protein area of the RD group divided by the area of the control group for proteins with FDR < 5%. A subset of proteins with immune function was identified by searching the protein function column with keywords “immune,” “inflam” “inflammation”, “cytokine”, “chemokine”, “macrophage”, “monocyte”, “T cell”, “neutrophil”, “TNF”, “nitric oxide”, “MHC”, “interferon”,” phagocytosis”, and “microglia.”

### Enzyme-Linked Immunosorbent Assay

MIF protein levels were measured in retina-vitreous protein extracts by a colorimetric sandwich ELISA kit (Mouse MIF DuoSet ELISA, R&D Systems, sensitivity range, 125–8000 pg/ml) per the manufacturer’s instructions. 96 well-plates were coated over night with the capture antibody, washed, blocked with blocking solution and incubated at room temperature for 1.5 hours. The biotinylated detection antibody was applied. Protein concentration was calculated from a standard curve of recombinant MIF. Samples were run in duplicate per assay. Equal amounts of extracted proteins determined by BCA assay in lysis buffer were loaded with lysis buffer per well. Samples were run in duplicate. The final results were expressed as the ratio of MIF protein expression in picograms per total protein in microgram.

### Immunohistochemistry (IHC)

Immunofluorescence was performed to detect markers as described^15^ using antibodies to glial fibrillary acidic protein (GFAP) (mouse monoclonal, Sigma-Aldrich), phospho-p44/42 MAPK(Erk1/2) (137F5, rabbit monoclonal, Cell Signaling), cleaved caspase-3 (Asp175, rabbit polyclonal, Cell Signaling), cleaved PARP (Asp214, mouse monoclonal, Cell Signaling), AIF (H-300, rabbit ployclonal, Santa Cruz Biotechnology), Sox-2 (Y-17, goat polyclonal, Santa Cruz Biotechnology), and arginase-1 (N-20, goat polyclonal, Santa Cruz Biotechnology). Slides were blocked with PBS containing 5% bovine serum albumin, and 1% Triton-X 100. Alexa Fluor 488 or 568 conjugated secondary antibodies (Invitrogen) were applied after 30 minute incubation with 100% normal goat serum. Omission of the primary antibody was used as a control for background staining; omission of primary and secondary antibodies was used as a control for autofluorescence. Cell nuclei were counterstained with DAPI (Invitrogen) and DRAQ5 (Thermo Scientific). Immunoperoxidase IHC was performed to evaluate macrophages and microglia using antibody to F4/80 (rat monoclonal, IgG2b, AbD Serotec), since autofluorescence was a confounding factor in the subretinal space. Goat IgG (Vector Laboratories) in blocking solution was applied to block Fc receptors. Secondary goat-anti-rat(Invitrogen) conjugated with horseradish peroxidase were applied. ImmPACT NovaRED kit (Vector Laboratories) was used to visualize the markers. Hematoxylin was used as a nuclear counterstain.

### TUNEL assay

TUNEL assay was performed to detect apoptosis and cell death say with the In Situ Cell Death Kit (TMR red; 1215679910, Roche Applied Science) per the manufacturer’s instructions.

### Western Immunoblotting Assay

Quantitated equal amount of total protein was run with a precast 4–20% Tris-glycine gel (Novex Wedge well, Invitrogen). Proteins on the gel were electroblotted onto a polyvinylidene fluoride membrane (Bio-Rad Laboratories). After blocking with 5% non fat dry milk, the membrane was immunoblotted for AIF expression. The blots were developed using SuperSignal™ West Femto Maximum Sensitivity Substrate (Thermo Scientific). The expression of α-tubulin (11H10, rabbit monoclonal, Cell Signaling) as a loading control was also evaluated after stripping off AIF antibody to normalize the data.

### Quantitative real-time (qRT)-PCR

qRT-PCR was performed as previously described^15^. Briefly, total RNA was extracted from individual retinas using TRIzol (Invitrogen). cDNA was synthesized (SuperScript VILO cDNA Synthesis Kit (Invitrogen)). Gene expression of murine *Nos2, Ccl2, Cxcl10, Arg1, Mrc1, Tnfa, Il1b*, and *Cd163* was quantified using catalogued Taqman probes and normalized to *Gapdh* (Applied Biosystems, ABI, Table S2).

### Image analysis

Photomicrographs were obtained using a Leica DM5000B fluorescent microscope and Leica DC500 digital camera, or a Nikon Eclipse 50i microscope and Nikon Digital Sight DS-U1 camera with NIS-Elements software for immunoperoxidase images. Confocal images were obtained using a Zeiss LSM 510 at the Hunt-Curtis Imaging Facility. Sections from at least five different animals at the area of detachment were evaluated. Treatment groups were analyzed in a masked fashion. To minimize the variability of region-specific differences within the retina, the same region of retina was evaluated for the fellow-eye control and RD areas. Identical illumination, microscope, and camera settings were used. Fixed areas were randomly sampled across all retinal layers in areas of maximal detachment or similar control retina areas. Peripapillary retina and ora serrata were excluded from analysis. The retina was selected as the region of interest from the 200X field of view. Image analysis was performed with ImageJ64 (ImageJ64, National Institutes of Health, Bethesda, MD, USA) as previously described^29^. For outer nuclear layer (ONL) thickness measurements, the ratio of ONL thickness to total retinal thickness was determined to minimize sectioning artefacts and artefacts from undulations in the retina induced by the RD. GFAP and pERK levels were quantitated, dividing the mean gray value of the selected retina by the retinal area in mm^2^. Figure images were optimized for color, brightness, and contrast. For macrophage quantitation and phenotypic analysis, hot-spots of F4/80 + cells were imaged at 200X magnification and counted per mm^2^ retina. The ratio of arginase-1 + to F4/80 + macrophages was determined per 200X field of view. For TUNEL analysis, cell counting was performed by using Image-Pro 6.2 (Media Cybernetics, Bethesda, MD, USA). Cells with immunofluorescence signal above the threshold within fixed threshold-designated retinal regions (measuring 0.047 mm^2^) in the ONL were counted. Counting was also performed using ImageJ, Retina Analysis Toolkit high-threshold macro, on cropped images of the entire ONL from a 900 × 900 pixel image to identify TUNEL-positive cells with signal above the threshold within fixed, threshold-designated retinal regions. Multichannel thresholding was performed to quantitate TUNEL-positive cells that also exhibited DAPI positivity.

### Statistics

Data were collected on standardized tables after analysis in a masked fashion. Error bars are listed with standard error of the mean. Student’s T-test was performed to evaluate the differences between two experimental groups, with p ≤ 0.05 considered statistically significant. In experiments with multiple groups, ANOVA was performed with Tukey post-hoc testing using JMP software.

### Study approval

This research adheres to the principles of the ARVO Statement for the Use of Animals in Ophthalmic and Vision Research. It was conducted under a protocol approved by our Institutional Animal Care and Use Committee.

## Electronic supplementary material

Supplementary Materials
