# Supplementary material for: MIF Inhibitor ISO-1 Protects Photoreceptors and Reduces Gliosis in Experimental Retinal Detachment
Source: Sci Rep. 2017 Oct 30;7:14336. doi: 10.1038/s41598-017-14298-9 (PMC5662618; doi:10.1038/s41598-017-14298-9)
Supplement: Supplementary file 1 — Supplementary Materials [file 41598_2017_14298_MOESM1_ESM.pdf]

MIF Inhibitor ISO-1 Protects Photoreceptors and Reduces Gliosis in Experimental Retinal Detachment

Bongsu Kim,[1] Rania Kusibati,[1] Tyler Heisler-Taylor,[1] Dimosthenis Mantopoulos,[1] Jiaxi Ding,[1] Mohamed H. Abdel-Rahman,[1,2,3] Abhay R. Satoskar,[4] Jonathan P. Godbout,[5] Sanjoy K. Bhattacharya,[6] and Colleen M. Cebulla[*1]

1. Havener Eye Institute, Department of Ophthalmology and Visual Science, The Ohio State University Wexner Medical Center, Columbus, OH 43212.
2. Division Human Genetics, The Ohio State University Wexner Medical Center, Columbus, OH 43240.
3. Department of Pathology, Menoufiya University, Egypt
4. Division Experimental Pathology, The Ohio State University Wexner Medical Center, Columbus, OH 43210.
5. Department of Neuroscience, The Ohio State University Wexner Medical Center, Columbus, OH 43210.
6. Bascom Palmer Eye Institute, Department of Ophthalmology, The Miller School of Medicine, Miami, FL 33136.

* Correspondence to

Colleen M. Cebulla, MD, PhD

915 Olentangy River Road, Suite 5000

Columbus, OH 43212

Phone 614-652-2600

Email: colleen.cebulla@osumc.edu

Supplementary Materials

Please see supplementary materials for complete iTRAQ protein list and macrophages polarization studies.

Figure S1. ISO-1 does not alter retinal macrophage polarization.

Table S1. iTRAQ Proteomic Data: All Proteins with FDR< 0.05

Table S2. PCR primers

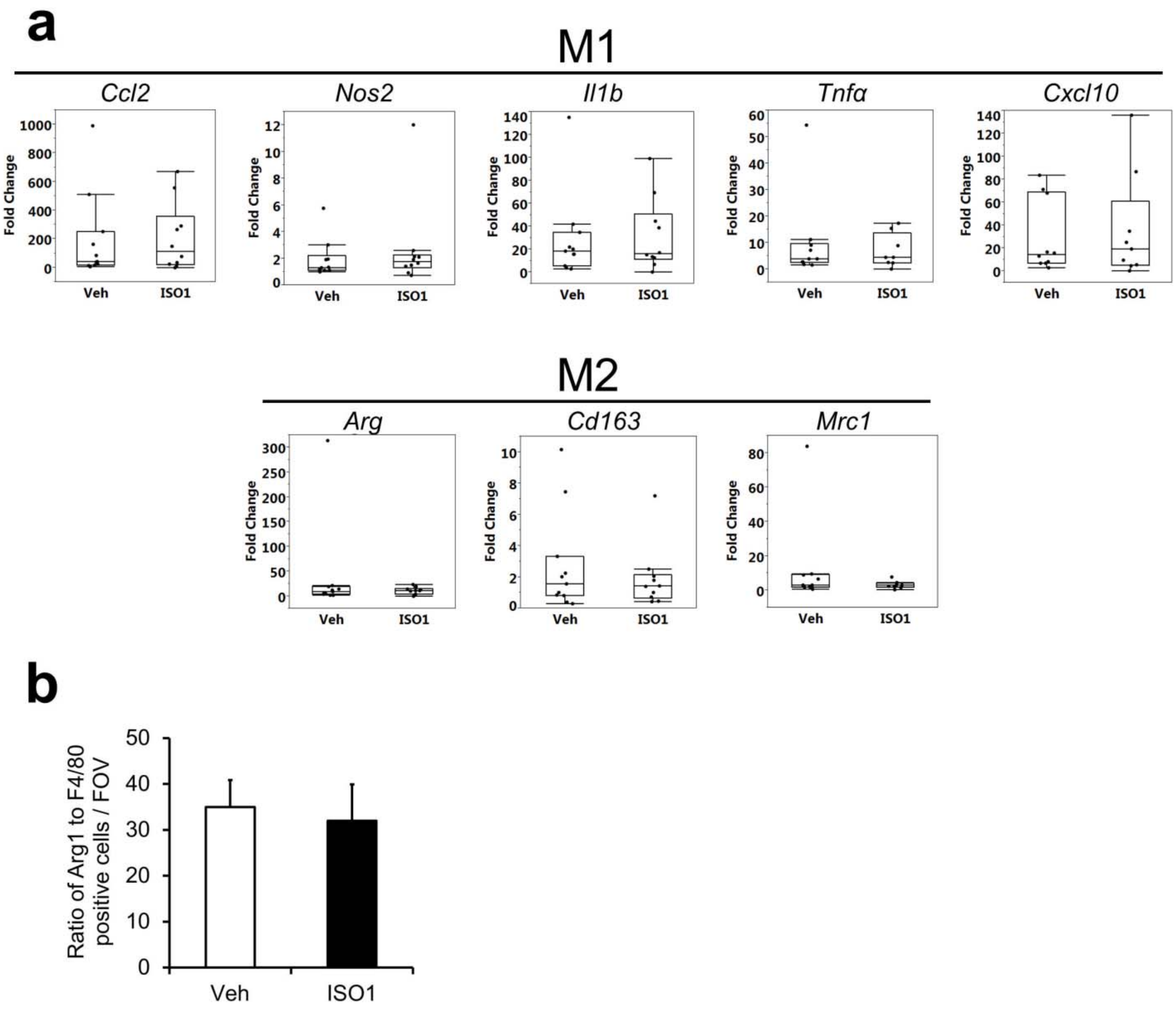

Figure S1. ISO-1 does not alter retinal macrophage polarization. (**a**) qPCR of gene expression in individual day 3 detached retinas (normalized to *Gapdh* and expressed as fold-change compared to fellow eye control retina) shows no significant difference in expression of specific M1 (*Ccl2* (p=0.45), *Nos2* (p=0.27), *Il1b* (p=0.42), *Tnfα* (p=0.69) and *Cxcl10* (p= 0.36)) or M2 (*Arg1* (p=0.82), *Cd163* (p=0.77) and *Mrc1* (p=0.85) genes with ISO-1 treatment. (**b**) Quantitation of Arg-1 positive to total (F4/80 positive) macrophages.

Table S1. iTRAQ Proteomic Data: All Proteins with FDR< 0.05

ALL PROTEINS SORTED BY DESCENDING 2 WEEK RATIO							
Swiss Protein #	Protein Name	MW (Kda)	Total Area in 2wk Treatment	Total Area in 4wk Treatment	Total Area in Control	2wk Ratio	4wk Ratio
P24622.1	Alpha-crystallin A chain	22.49	24181.09	0	0	Undef*	Undef
P61089.1	Ubiquitin-conjugating enzyme E2N	17.14	10199.02	8319.79	0	Undef	Undef
P26350.2	Prothymosin alpha	12.26	9594.33	0	0	Undef	Undef
P63044.2	Vesicle-associated membrane protein 2	12.69	6123.64	4889.5	0	Undef	Undef
Q99LF4.1	UPF0027 protein C22orf28 homolog	55.26	5582.83	6450.06	0	Undef	Undef
Q9WVJ5.3	Beta-crystallin B1	28.01	5042.3	0	0	Undef	Undef
P04342.2 Q03740.4 Q9CXV3.3 P04344.3 P04345.2 Q61597.3	Gamma-crystallin D	21.01	3924.47	810.78	0	Undef	Undef
O35633.3	Vasicular inhibitory amino acid transporter 10D	57.39	3745.41	6105.73	0	Undef	Undef
P14231.2	ATPase, Na+/K+ transporting, beta 2 polypeptide	33.35	3561.26	5832.68	0	Undef	Undef
Q148R9.2	Regulator of G-protein signalling 9 binding protein	25.18	2672.02	2502.36	0	Undef	Undef
P62751.1	60S ribosomal protein L23a	17.7	2426.28	901.37	0	Undef	Undef
Q6NVF9.1	Cleavage and polyadenylation specificity factor subunit 6	59.16	2414.83	1884.92	0	Undef	Undef
P80318.1	Chaperonin containing Tcp1, subunit 3 (gamma)	60.64	1794.38	369.22	0	Undef	Undef
Q9Z275.3	Retinaldehyde-binding protein 1	36.42	1443.89	3084.9	0	Undef	Undef
O88746.1 Q5SRX1.1	Target of myb1-like 2 (chicken)	54.33	1428.39	732.34	0	Undef	Undef
Q9DCS9.3	NADH dehydrogenase (ubiquinone) 1 beta subcomplex subunit 10	21.03	1236.96	1125.06	0	Undef	Undef
P28271.2 Q811J3.1	Aconitase 1	98.19	1062.83	626.63	0	Undef	Undef
EDL33110.1	Splicing factor 3b, subunit 2	98.22	1047.71	836.13	0	Undef	Undef
P24288.2	Branched-chain-amino-acid aminotransferase, cytosolic	42.8	1015.04	0	0	Undef	Undef
Q9D8B3.2	Chromatin modifying protein 4B	24.94	942.19	623.8	0	Undef	Undef
P50518.2	Vacuolar proton pump subunit E 1	26.16	928.31	2121.26	0	Undef	Undef
P60710.1 P63260.1	Actin, cytoplasmic 1	41.74	748.35	2877.89	0	Undef	Undef
P01942.2 P06467.2	Hemoglobin subunit alpha	15.09	732.27	1548.91	0	Undef	Undef
P09602.2	Non-histone chromosomal protein HMG-17	9.42	713.04	943.38	0	Undef	Undef
Q02053.1	Ubiquitin-like modifier activating enzyme 1	117.83	578.58	0	0	Undef	Undef
Q62167.3	Embryonic RNA helicase	73.11	480.42		0	Undef	Undef
Q9QW08.1	Phosducin	28.02	444.13	976.64	0	Undef	Undef
Q3UIU2.3	NADH dehydrogenase [ubiquinone] 1 beta subcomplex subunit 6	15.52	295.38	314.9	0	Undef	Undef
O70251.5	Eukaryotic translation elongation factor 1 beta 2	24.7	280.17	796.24	0	Undef	Undef

Q9CQX2.1	Cytochrome b5 type B	16.32	253.83	0	0	Undef	Undef
Q9EQF6.1	Dihydropyrimidinase-related protein 5	61.25	237.25	0	0	Undef	Undef
P62696.2	Beta-crystallin B2	23.39	56113.62	0	1700.19	33.00	0.00
Q9JJU9.3	Beta-crystallin B3	24.3	12504.74	1626.71	1028.54	12.16	1.58
P62204.2	Calmodulin	16.84	8826.97	8055.44	785.11	11.24	10.26
P08228.2	Superoxide dismutase 1	15.95	4132.91	2269.88	492.25	8.40	4.61
Q62318.3	Transcriptional corepressor TIF1beta	82.83	2768.9	0	337.13	8.21	0.00
P23927.2	Alpha-crystallin B chain	20.07	56323.52	2261.05	7173.68	7.85	0.32
O54879.3	High mobility group protein B3	23.01	2451.69	840.39	315.29	7.78	2.67
Q9JJV1.3	Beta-crystallin A2	22.24	28616.83	2018.08	3726.7	7.68	0.54
Q61029.3	Lamina-associated polypeptide 2, isoforms beta/delta/epsilon/gamma	50.3	8912.23	6426.06	1193.33	7.47	5.38
Q9JJV0.3	Beta-crystallin A4	22.47	30635.81	0	4182.34	7.33	0.00
Q61656.1	RNA helicase p68	69.33	3161.17	744.32	463.87	6.81	1.60
P63028.1	Tumor protein, translationally-controlled 1	19.46	11030.75	10353.32	1781.43	6.19	5.81
P48193.2	Erythrocyte protein band 4.1	95.93	7957.56	1637.76	1291.39	6.16	1.27
P46471.5	Proteasome 26S subunit ATPase 2	48.66	9047.89	5672.95	1518.83	5.96	3.74
O35737.3<br>P70333.1	Heterogeneous nuclear ribonucleoprotein H1	49.21	14635.49	11500.97	2463.04	5.94	4.67
Q8BG05.1<br>P49312.2	Heterogeneous nuclear ribonucleoprotein HA3	39.66	2206.54	444.11	387.96	5.69	1.14
Q9JIQ3.1	Diablo homolog (Drosophila)	26.84	2589.72	821.89	461.29	5.61	1.78
P14094.1	Sodium/potassium-dependent ATPase<br>subunit beta-1	35.2	2447.73	1632.09	444.79	5.50	3.67
Q59J78.1	Mimitin, mitochondrial	19.63	4217.82	2634.77	779.78	5.41	3.38
P12787.2	Cytochrome c oxidase subunit 5A, mitochondrial	16.1	3532.55	4748.54	658.9	5.36	7.21
Q9WVT6.1	Carbonic anhydrase 14	37.51	2761.02	1121.98	522.21	5.29	2.15
Q8BL97.1	Serine/arginine-rich splicing factor 7	30.82	1799.83	1104	340.97	5.28	3.24
O08539.1	Myc box-dependent-interacting protein 1	64.48	8092.18	2841.11	1543.61	5.24	1.84
Q61597.3	Gamma-crystallin C	20.92	212073.68	14072.34	40904.88	5.18	0.34
Q9JM96.1	Cdc42 effector protein 4	37.87	17089.85	17928.37	3379.61	5.06	5.30
Q91WU5.1	Arsenite methyltransferase	41.8	1673.88	185.21	339.92	4.92	0.54
O35486.3	Beta-crystallin S	20.85	23553.22	2388.94	4819.85	4.89	0.50
Q5SUR0.1	Phosphoribosylformylglycinamidine synthase	144.65	1949.82	1322.24	403.35	4.83	3.28
Q6IRU2.3	Tropomyosin 4	28.47	2041.69	2834.1	424.82	4.81	6.67
P52480.4	Pyruvate kinase muscle isozyme	57.85	6396.53	5781.34	1351.92	4.73	4.28
P02088.2<br>P02089.2<br>P02104.2<br>P04444.3<br>P04443.5	Hemoglobin subunit beta-1	15.84	426079.84	246470.22	91563.03	4.65	2.69
P06151.3<br>P16125.2<br>P00342.2	L-lactate dehydrogenase A chain	36.5	2836.82	2244.26	609.98	4.65	3.68
Q8R1W8.1	Interphotoreceptor matrix proteoglycan 1	89.49	8176.43	7044.2	1792.02	4.56	3.93

P04344.3	Gamma-crystallin B	21.14	102221.95	6393.76	22605.57	4.52	0.28
P61982.2	Tyrosine 3-monooxygenase/tryptophan 5-monooxygenase activation protein, gamma polypeptide	28.31	6918.93	1979.98	1575.31	4.39	1.26
P08752.4	G protein Gi2 alpha	40.48	2877.17	1473.3	656.43	4.38	2.24
Q5XG69.2	Fam169a protein	73.27	1259.76	1700.35	293.81	4.29	5.79
Q91VR2.1	ATP synthase subunit gamma, mitochondrial	32.89	3996.72	4491.29	965.86	4.14	4.65
P20612.3	Rod-type transducin alpha subunit	39.97	3678.58	2098.15	897.18	4.10	2.34
Q7TQF7.1	Amphiphysin	75.03	6751.83	5128.86	1655.48	4.08	3.10
P02088.2	Hemoglobin subunit beta-1	15.84	69331.96	31995.95	17292.18	4.01	1.85
O35326.1	Serine/arginine-rich splicing factor 5	30.95	3617.17	1153.41	915.56	3.95	1.26
Q00915.2	Retinol-binding protein 1	15.85	5865.27	6213.71	1493.18	3.93	4.16
P11031.3	SUB1 homolog	14.43	9495.94	7053.68	2441.04	3.89	2.89
O54983.1	Mu-crystallin homolog	33.53	5676.2	2669.46	1461.76	3.88	1.83
P63325.1	40S ribosomal protein S10	18.92	26719.4	17879.76	7091.96	3.77	2.52
Q9DB20.1	ATP synthase subunit O, mitochondrial	23.37	8835.4	4135.24	2363.55	3.74	1.75
Q3U0V1.1	Far upstream element-binding protein 2	76.83	9987.53	9289.15	2673.77	3.74	3.47
Q99L13.1	3-hydroxyisobutyrate dehydrogenase, mitochondrial	35.44	5704.01	4339.65	1547.98	3.68	2.80
P02525.1	Beta-crystallin A1	23.41	84148.33	31972.28	22944.86	3.67	1.39
P99028.2	Cytochrome b-c1 complex subunit 6, mitochondrial	10.44	5542.12	4216.49	1524.25	3.64	2.77
P12382.3	Phosphofructokinase 1	85.31	3964.67	2360.7	1095.33	3.62	2.16
P35700.1	Peroxiredoxin 1	22.18	12360.24	14324.8	3415.58	3.62	4.19
P35564.1	Calnexin	67.28	2839.24	2498.99	830.31	3.42	3.01
Q9D8Y0.1	EF hand domain containing 2	26.8	5306.37	3327.7	1566.48	3.39	2.12
P24549.5	Retinal dehydrogenase 1	54.48	2259.61	3049.02	677.39	3.34	4.50
P16858.2	Glyceraldehyde-3-phosphate dehydrogenase	35.81	8088.75	7873.62	2433.15	3.32	3.24
P80313.1	T-complex protein 1 subunit eta	59.66	5315.66	4406.01	1610.78	3.30	2.74
Q9D1K2.2	V-type proton ATPase subunit F	13.37	16079.2	16065.27	4878.29	3.30	3.29
Q6PHZ2.1	Calcium/calmodulin-dependent protein kinase type II delta chain	56.38	6032.79	4640.37	1850.78	3.26	2.51
Q64523.3	Histone H2A type 2C	13.99	8447.43	7682.35	2639.83	3.20	2.91
P50516.2	V-type proton ATPase catalytic subunit A	68.33	1512.01	2390.21	473.94	3.19	5.04
Q99JF8.1	PC4 and SFRS1 interacting protein 1	59.71	18685.6	14880.96	5922.72	3.15	2.51
Q9JIX8.2	Apoptotic chromatin condensation inducer in the nucleus	150.72	26402.33	15840.9	8565.74	3.08	1.85
P97370.1	Sodium/potassium-transporting ATPase subunit beta-3	31.78	9221.83	7903.77	3054.73	3.02	2.59
Q60668.2	Heterogeneous nuclear ribonucleoprotein D0	38.36	18349.77	17466.82	6110.31	3.00	2.86
Q9CZW5.1	Translocase of outer mitochondrial membrane 70 homolog A (yeast)	67.53	10448.75	7460.74	3534.36	2.96	2.11
P20444.3	Protein kinase C, alpha	76.86	20419.48	17117.23	7025.37	2.91	2.44

Q7TMM9.1	Tubulin beta-2A chain	49.92	29009.67	22038.55	9996.3	2.90	2.20
Q9CSU0.2	Cell cycle-related and expression-elevated protein in tumor	36.89	793.01	941.84	277.3	2.86	3.40
Q91YT0.1	NADH-ubiquinone oxidoreductase 51 kDa subunit	50.84	1638.27	1463.66	574.13	2.85	2.55
P62259.1	Tyrosine 3-monooxygenase/tryptophan 5-monooxygenase activation protein, epsilon polypeptide	29.18	4290.64	1638.96	1509.37	2.84	1.09
P99026.1	Proteasome subunit, beta type 4	28.99	2389.68	4115.01	862.11	2.77	4.77
Q04447.1	Creatine kinase B-type	42.72	9014.48	10564.06	3274.85	2.75	3.23
Q52KI8.1	Serine/arginine repetitive matrix protein 1	106.91	29163.71	16306.29	10844.67	2.69	1.50
P10630.2	Protein synthesis initiation factor 4A	46.41	16616.88	15916.03	6200.09	2.68	2.57
Q8R1B5.1	Complexin-3	10.37	44258.38	26332.53	16515.56	2.68	1.59
Q501J6.1	Probable ATP-dependent RNA helicase DDX17	72.41	4192.48	1970.21	1565.96	2.68	1.26
P19536.1	Cytochrome c oxidase polypeptide Vb	13.82	3967.98	1742.22	1495.72	2.65	1.16
P47962.3	60S ribosomal protein L5	34.41	12134.49	11293.41	4616.95	2.63	2.45
Q9CRB9.1	Coiled-coil-helix-coiled-coil-helix domain-containing protein 3, mitochondrial	26.34	10975.34	6715.59	4204.49	2.61	1.60
P31648.2	Sodium- and chloride-dependent GABA transporter 1	67.01	89511.63	77110.21	34395.34	2.60	2.24
P30681.3	High mobility group protein B2	24.16	26162.88	18308.46	10096.6	2.59	1.81
P31650.1	Sodium- and chloride-dependent GABA transporter 3	69.9	9212.58	8175.16	3562.6	2.59	2.29
P70404.1	Isocitric dehydrogenase subunit gamma	42.79	6460.35	4468.21	2507.81	2.58	1.78
Q8BLK3.1	Limbic system-associated membrane protein	38.09	2335.72	4428.68	906.76	2.58	4.88
Q9CQ89.3	Brain acetylcholinesterase putative membrane anchor	18.87	4554.5	3665.98	1778.28	2.56	2.06
Q00623.1	Apolipoprotein A-I	30.6	4624.76	848.32	1807.95	2.56	0.47
Q8K003.1	Coiled-coil domain-containing protein 72	7.07	29252.97	18895.06	11467.98	2.55	1.65
Q8CIE6.1	Coatomer protein complex subunit alpha	138.47	6494.61	6601.88	2559.77	2.54	2.58
Q99PT1.3	Rho GDP dissociation inhibitor 1	23.41	6615.84	7648.32	2610.02	2.53	2.93
P49722.2	Proteasome subunit alpha type-2	25.93	7557.74	4923.28	2994.9	2.52	1.64
P07724.3 P02772.1	Serum albumin	68.7	6082.72	3371.51	2412.8	2.52	1.40
P10637.3	Microtubule-associated protein tau isoform b	76.26	17878.29	11214.5	7105.01	2.52	1.58
Q9QZ06.1	Toll interacting protein	30.35	8038.4	5694.64	3195.89	2.52	1.78
O08795.1	Glucosidase 2 subunit beta	58.8	12484.76	8407.29	4968.34	2.51	1.69
Q99020.1	Heterogeneous nuclear ribonucleoprotein A/B	30.84	12461.34	14428.6	4969.93	2.51	2.90
P10107.2	Annexin A1	38.74	6665.01	3027.56	2674.5	2.49	1.13
P18872.3	Guanine nucleotide binding protein, alpha o isoform A	40.09	44841.72	43398.99	18024.27	2.49	2.41
P57780.1	Non-muscle alpha-actinin 4	105	18083.9	15843.58	7342.45	2.46	2.16
Q8BGD9.1	Eukaryotic translation initiation factor 4B	68.85	8746.16	7346.48	3561.58	2.46	2.06

P00405.1	Cytochrome C oxidase subunit 2	25.98	9366.25	17373.49	3814.8	2.46	4.55
Q8BP92.1	Reticulocalbin 2	37.28	3243.42	2578.68	1321.1	2.46	1.95
Q9WTT4.1	V-type proton ATPase subunit G 2	13.66	2935.26	2897.78	1217.26	2.41	2.38
P47708.2	Rabphilin 3A	75.5	7923.63	10194.69	3312.19	2.39	3.08
P16858.2	Glyceraldehyde-3-phosphate dehydrogenase	35.81	26452.97	11145.87	11147.95	2.37	1.00
Q60972.4	Retinoblastoma-binding protein 4	47.66	13005.98	9899.45	5486.43	2.37	1.80
P20029.3	Immunoglobulin heavy chain-binding protein	72.43	8349.11	6081.91	3542.65	2.36	1.72
P26443.1	Glutamate dehydrogenase 1	61.34	6555.27	5726.4	2795.8	2.34	2.05
Q9WV55.2	Vesicle-associated membrane protein,<br>associated protein A	27.86	18811.09	8761.16	8043.72	2.34	1.09
P17710.2	Hexokinase 1	108.32	1297.1	3088.18	557.36	2.33	5.54
O35215.3	D-dopachrome decarboxylase	13.08	8751.73	5141.54	3769.93	2.32	1.36
Q62093.4	Serine/arginine-rich splicing factor 2	25.48	2618.81	1143.24	1132.99	2.31	1.01
Q7TNV0.1	Protein DEK	43.16	11194.6	9253.78	4852.17	2.31	1.91
P35803.2	Neuronal membrane glycoprotein M6-b	36.22	18660.55	17096.86	8096.68	2.30	2.11
Q9D0M3.1	Cytochrome c1, heme protein, mitochondrial	35.33	7242.41	6179.25	3147.02	2.30	1.96
P08113.2	Endoplasmin	92.49	4607.99	3653.32	2002.84	2.30	1.82
P63158.2	High mobility group protein B1	24.9	50850.31	31276.46	22140.23	2.30	1.41
P61264.1	Syntaxin 1B	33.25	12346.72	7530.91	5396.87	2.29	1.40
Q80TB8.2	Synaptic vesicle membrane protein VAT-1 homolog-like	45.82	20176.46	13038.15	8866.82	2.28	1.47
P16546.4	Alpha-II spectrin	284.66	4373.8	1551.39	1933.09	2.26	0.80
Q9Z273.1	Tubby like protein 1	60.34	11038.76	5107.89	4886.93	2.26	1.05
P46935.3	Ubiquitin-protein ligase NEDD4	102.72	6311.79	7798.78	2803.24	2.25	2.78
BAE37215.1	Unnamed protein product	16.15	2499.4	0	1110.53	2.25	0.00
Q62261.2	Spectrin beta chain, brain 1	274.28	145790.14	99552.82	64798.08	2.25	1.54
Q06185.2	ATP synthase subunit e, mitochondrial	8.24	25367.74	21354.29	11291.78	2.25	1.89
P51859.2	Hepatoma derived growth factor	26.27	38651.42	29131.4	17265.4	2.24	1.69
P23198.2	Chromobox protein homolog 3	20.86	4492.49	0	2010.43	2.23	0.00
Q9D0J8.3	Parathymosin	11.43	17494.31	13805.77	7838.87	2.23	1.76
P46096.1	Synaptotagmin 1	47.42	12967.6	14022.08	5879.26	2.21	2.39
P26041.3	Moesin	67.78	8591.16	8244.09	3897.31	2.20	2.12
Q9JHU4.1	Cytoplasmic dynein 1 heavy chain 1	532.13	11589.4	6774.75	5260.89	2.20	1.29
Q9R1P4.1	Proteasome subunit alpha type 1	29.55	8932.56	8932.54	4057.54	2.20	2.20
XP_983229.1	40S ribosomal protein S12-like	14.49	25581.32	25048.77	11625.69	2.20	2.15
Q60597.3	2-oxoglutarate dehydrogenase complex<br>component E1	116.47	30432.13	26448.5	13836.56	2.20	1.91
Q91X97.4	Neurocalcin-delta	22.25	8524.84	10236.39	3879.69	2.20	2.64
Q9Z2X1.3	Heterogeneous nuclear ribonucleoprotein F	45.74	2405.12	3871.37	1094.81	2.20	3.54
O88271.1	Craniofacial development protein 1	32.81	22268.32	18647.22	10154.63	2.19	1.84
Q91ZZ3.1	Beta-synuclein	14.05	153461.33	114748.73	70060.86	2.19	1.64

Q9R0P4.1	Small acidic protein	45.74	11932.3	8909.65	5464.29	2.18	1.63
P49817.1	Caveolin-1	20.54	17735.57	15737.36	8137.56	2.18	1.93
Q9DCT2.2	NADH dehydrogenase [ubiquinone] iron-sulfur protein 3, mitochondrial	30.15	10435.59	11112.37	4801.2	2.17	2.31
Q91V61.1	Sideroflexin-3	35.41	3125.14	1523.69	1439.86	2.17	1.06
O35381.1	Acidic leucine-rich nuclear phosphoprotein 32 family member A	28.54	17615.64	16772.23	8127.26	2.17	2.06
Q9CZ13.1	Cytochrome b-c1 complex subunit 1, mitochondrial	52.78	10544.87	7038.68	4881.99	2.16	1.44
Q99P72.2	Neurite outgrowth inhibitor	126.63	4190.71	2040.9	1942.5	2.16	1.05
P31786.2	Acyl-CoA-binding protein	10	20496.25	19182.33	9501.31	2.16	2.02
P17742.2	Peptidyl-prolyl cis-trans isomerase A	17.97	17037.9	10064.62	7901.16	2.16	1.27
Q91WJ8.1	Far upstream element (FUSE) binding protein 1	68.56	36445.18	35249.27	16907.51	2.16	2.08
Q9R0P9.1	Ubiquitin carboxyl-terminal hydrolase isozyme L1	24.84	68304.19	64010.88	31717.59	2.15	2.02
P47753.4	F-actin-capping protein subunit alpha-1	32.95	35736.42	21495.29	16630.5	2.15	1.29
Q8K183.1	Pyridoxal kinase	35.02	11708.58	10429.7	5450.76	2.15	1.91
P62702.2	40S ribosomal protein S4, X isoform	29.6	10041.52	8398.14	4684.02	2.14	1.79
Q61644.1	Protein kinase C and casein kinase substrate in neurons 1 isoform 1	50.59	21769.08	16839.72	10177.01	2.14	1.65
Q64704.1	Syntaxin 3A	33.25	10154.43	7554.3	4757.11	2.13	1.59
Q80XU3.1	Nuclear ubiquitous casein and cyclin-dependent kinases substrate	26.32	20342.2	18885.12	9548.2	2.13	1.98
P52293.2	Importin alpha P1	57.94	6751.75	9184.2	3169.95	2.13	2.90
Q9D710.1	Thioredoxin-related transmembrane protein 2	33.95	12466.04	8804.2	5871.23	2.12	1.50
P18760.3	Cofilin-1	18.56	27050.93	27944.34	12757.07	2.12	2.19
Q9DC69.1	NADH dehydrogenase [ubiquinone] 1 alpha subcomplex	42.51	3992.3	1653.11	1887.02	2.12	0.88
Q9CT10.2	Ran-binding protein 3	52.58	11029.31	6761.14	5213.25	2.12	1.30
Q8R0A7.1	Uncharacterized protein KIAA0513	46.33	40821.59	40997.04	19306.24	2.11	2.12
P46460.2	Vesicle-fusing ATPase	82.63	28656.55	26301.71	13646.02	2.10	1.93
P62270.3	40S ribosomal protein S18	17.72	9269.96	6746.84	4415.25	2.10	1.53
P11352.2	Glutathione peroxidase 1	22.18	9088.67	8650.58	4346.3	2.09	1.99
Q9DBJ1.3	Phosph oglycerate mutase 1	28.84	25567.69	21260.43	12272.12	2.08	1.73
P15864.2	Histone H1.2	21.27	19647.88	22139.96	9435.2	2.08	2.35
Q9Z2W0.1	Aspartyl aminopeptidase	52.17	12652.62	8069.76	6089.63	2.08	1.33
P14152.3	Malate dehydrogenase, cytoplasmic	36.52	26915.12	18580.71	12959.36	2.08	1.43
P48722.2	Heat shock 70 kDa protein 4L	94.4	1423.67	3492.55	687.8	2.07	5.08
Q91WQ3.3	Tyrosyl-tRNA synthetase, cytoplasmic	59.11	14438.23	12228.33	6987.03	2.07	1.75
P12367.2	cAMP-dependent protein kinase type II-alpha regulatory subunit	45.4	10968.92	8019.57	5345.68	2.05	1.50
Q9D6J5.1	NADH dehydrogenase [ubiquinone] 1 beta subcomplex	21.88	85163.7	76199.85	41604.85	2.05	1.83
Q9D8N0.3	Elongation factor 1-gamma	50.07	14973.95	13693.64	7328.63	2.04	1.87
Q9D8W5.3	26S proteasome non-ATPase regulatory subunit 12	52.89	2040.16	1542.66	1001.51	2.04	1.54

Q9D1D4.1	Transmembrane emp24 domain-containing protein 10	24.91	26131.54	28220.69	12861.44	2.03	2.19
P51150.2	Ras-related protein Rab-7a	23.49	19644.83	16384.81	9671.15	2.03	1.69
P97797.1	Tyrosine-protein phosphatase non-receptor type substrate 1	56.43	71413.48	57840.74	35188.13	2.03	1.64
P63054.2	Purkinje cell protein 4	6.81	61584.66	48910.5	30402.27	2.03	1.61
P0CG50.1	Polyubiquitin-C	82.56	21790.16	16924.85	10830.92	2.01	1.56
Q9WUL7.1	ADP-ribosylation factor-like protein 3	20.49	10450.59	11886.85	5214.42	2.00	2.28
P63280.1	Ubiquitin-protein ligase I	18.01	48897.98	44915.38	24437.56	2.00	1.84
P43274.2	Histone H1.4	21.98	21633.45	17193.17	10823.97	2.00	1.59
Q8BH59.1	Calcium-binding mitochondrial carrier protein Aralar1	74.58	4783.69	7874.89	2394.67	2.00	3.29
Q03958.1	Prefoldin subunit 6	14.46	11900.85	7857.78	5960.63	2.00	1.32
P20152.3	Vimentin	53.7	41449.78	32913.89	20784.81	1.99	1.58
Q9R1Q8.1	Transgelin-3	22.48	69829.04	51916.29	35198.74	1.98	1.47
P20065.1	Thymosin beta-4	5.68	23584.77	23226.22	11910.68	1.98	1.95
Q61147.2	Ceruloplasmin	121.17	33196.12	27672.44	16774.19	1.98	1.65
Q68FD5.3	Clathrin heavy chain 1	191.59	30073.01	27019.77	15309.69	1.96	1.76
O70493.1	Sorting nexin-12	19.12	686.1	0	350.24	1.96	0.00
Q9EQU5.1	Phosphatase 2A inhibitor	33.38	5218.94	3714.61	2666.81	1.96	1.39
O08997.1	Copper transport protein ATOX1	7.34	16926.39	13795.21	8679.53	1.95	1.59
Q9DCG9.1	tRNA methyltransferase 112 homolog	14.14	2933.51	2278.27	1504.91	1.95	1.51
Q9CQ69.3	Cytochrome b-c1 complex subunit 8	9.77	74746.83	80168.45	38373.88	1.95	2.09
BAD32303.1	U5 snRNP-specific protein, 200 kDa	218.46	3063.08	2424.85	1572.61	1.95	1.54
Q9JMG7.2	Hepatoma-derived growth factor-related protein 3	22.43	28653.24	18937.84	14790.13	1.94	1.28
Q9JMF3.1	Guanine nucleotide-binding protein G(I)/G(S)/G(O) subunit gamma-13	7.98	21028.57	26516.14	10898.77	1.93	2.43
P61957.1	Sentrin-2	10.87	52827.5	39148.29	27390.14	1.93	1.43
Q9D6J6.2	NADH dehydrogenase [ubiquinone] flavoprotein 2, mitochondrial	27.29	106751.46	102979.25	55413.42	1.93	1.86
P14148.2	60S ribosomal protein L7	31.42	19798.92	17053.22	10345.53	1.91	1.65
P80315.3	T-complex protein 1 subunit delta	58.08	9382.08	9893.17	4903.91	1.91	2.02
XP_908833.1	PREDICTED: hypothetical protein	9.17	6028.88	5151.2	3154.36	1.91	1.63
P34884.2	Macrophage migration inhibitory factor	12.51	18097.33	15500.36	9499.3	1.91	1.63
Q9CQI6.3	Coactosin-like protein	15.95	46954.08	45392.29	24665.27	1.90	1.84
P70349.3	Histidine triad nucleotide-binding protein 1	13.78	43688.86	34949.41	22967.74	1.90	1.52
Q62376.2	U1 small nuclear ribonucleoprotein 70 kDa	52	19556.59	14084.23	10283.12	1.90	1.37
Q8VIJ6.1	Polypyrimidine tract-binding protein-associated-splicing factor	75.46	39939.22	35071.67	21038.17	1.90	1.67
Q01853.4	Transitional endoplasmic reticulum ATPase	89.33	17116.24	13365.85	9016.46	1.90	1.48
Q62277.2	Synaptophysin	34.03	12647.27	10614.29	6678.26	1.89	1.59
P62880.3	G protein subunit beta-2	37.34	1119.2	1084.35	592.44	1.89	1.83
Q9D855.3	Cytochrome b-c1 complex subunit 7	13.53	1087.01	2974.89	575.81	1.89	5.17
Q61012.3	Transducin gamma chain	8.53	47134.33	27350.48	24968.48	1.89	1.10

Q8CAQ8.1	Mitochondrial inner membrane protein	83.92	15735.29	14865.82	8337.12	1.89	1.78
Q920Q6.1	RNA-binding protein Musashi homolog 2	36.94	1864.22	3017.86	992.9	1.88	3.04
Q8BLQ9.2	Cell adhesion molecule 2	47.57	14663.47	12199.77	7810.85	1.88	1.56
P38647.2	Peptide-binding protein 74	73.54	54364.26	38369.39	28964.07	1.88	1.32
Q9D6F9.3	Tubulin beta-4 chain	49.59	12066.33	10843.03	6452.26	1.87	1.68
P97300.3	Neuroplastin	44.38	5595.54	5651.95	3002.91	1.86	1.88
P54227.2	Stathmin	17.28	13359.48	9304.18	7169.57	1.86	1.30
P14685.2	26S proteasome non-ATPase regulatory subunit 3	60.71	1661.24	1442.63	891.73	1.86	1.62
P50096.1 P24547.2	Inosine 5'-phosphate dehydrogenase 1	55.3	17257.56	15977.3	9276.68	1.86	1.72
Q7TSJ2.2	Microtubule-associated protein 6	96.47	23470.8	19422.44	12620.64	1.86	1.54
Q6ZWY9.3	Histone H2B type 1-C/E/G	13.91	27152.88	22907.85	14621.14	1.86	1.57
O55131.1	Histone H2B type 1	13.91	24815.05	23585.05	13363.01	1.86	1.76
Q9WUK2.3	Septin-7	50.56	34754.01	21874.84	18750.79	1.85	1.17
Q8R5M8.2	Synaptic cell adhesion molecule	49.8	38995.5	35280.41	21059.72	1.85	1.68
P84104.1	Serine/arginine-rich splicing factor 3	19.33	22595.27	18262.77	12273.86	1.84	1.49
P56480.2	ATP synthase subunit beta, mitochondrial	56.31	14904.78	12004.18	8127.91	1.83	1.48
P14873.1	Microtubule-associated protein 1B	270.45	7455.59	3066.67	4084.43	1.83	0.75
P10605.2	Cathepsin B	37.28	9542.57	9508.83	5236.01	1.82	1.82
P15532.1	Non-metastatic cells 1, protein	17.21	46204.19	46121.48	25368.7	1.82	1.82
Q5SUF2.1	Luc7-like protein 3	51.46	27012.38	31168.24	14850.31	1.82	2.10
P26645.2	Myristoylated alanine-rich C-kinase substrate	29.67	55104.61	55970.03	30349.39	1.82	1.84
Q9WUR9.1	Adenylate kinase isoenzyme 4, mitochondrial	25.07	5276.74	3011.77	2907.46	1.81	1.04
O35295.3	Transcriptional activator protein Pur-beta	33.91	14033.17	12211.29	7796.52	1.80	1.57
P84086.1	Serine/arginine-rich splicing factor 3	19.33	7502.4	4899.99	4181.97	1.79	1.17
P28652.2	Calcium/calmodulin-dependent protein kinase type II subunit beta	60.47	3732.65	4002.57	2088.98	1.79	1.92
Q9CWF2.1	Tubulin beta-2B chain	49.96	10357.28	9040.29	5797.65	1.79	1.56
P39053.2	Dynamin 1	91.72	22530.46	26063.14	12692.15	1.78	2.05
Q9CQI7.1	U2 small nuclear ribonucleoprotein B	25.33	5270.26	6128.46	2983.55	1.77	2.05
Q8R0A5.2	Transcription elongation factor A (SII)-like 3	22.47	6879.23	8330.04	3904.74	1.76	2.13
P56959.1	RNA-binding protein FUS	52.69	19283.12	29711.55	10951.47	1.76	2.71
P42932.3	Chaperonin containing Tcp1, subunit 8 (theta)	59.56	9795.18	4975.56	5570.11	1.76	0.89
Q6PIC6.1	Sodium/potassium-transporting ATPase subunit alpha-3	111.71	41780.86	47943.94	23815.83	1.75	2.01
P56135.3	ATP synthase subunit f, mitochondrial	10.34	42151.19	38903.19	24057.29	1.75	1.62
P11499.2	Heat shock protein HSP 90-beta	83.34	21465.34	23544.34	12258.12	1.75	1.92
Q91VR5.1	ATP-dependent RNA helicase DDX1	82.51	32706.96	26858.72	18696.5	1.75	1.44
A2ASS6.1	Titin	3906.95	66578.7	63552.6	38074.62	1.75	1.67
P62827.3	GTP-binding nuclear protein Ran	24.43	10128.7	9529.51	5794.65	1.75	1.64

Q99LY9.3	NADH dehydrogenase [ubiquinone] iron-sulfur protein 5;	12.65	6317.35	6256.09	3617.94	1.75	1.73
Q8CHC4.3	Synaptojanin-1	172.65	31978.7	23194.96	18497.19	1.73	1.25
Q60932.3	Outer mitochondrial membrane protein porin 1	32.36	98276.57	133282.64	56922.64	1.73	2.34
P13595.3	Neural cell adhesion molecule 1	119.44	4755.41	5540.99	2764.41	1.72	2.00
Q61316.1	Heat shock 70 kDa protein 4	94.15	7990.89	7720.77	4646.28	1.72	1.66
Q60864.1	Stress-induced-phosphoprotein 1	62.59	11414.24	10953.5	6664.58	1.71	1.64
Q9JKK7.2	Tropomodulin-2	39.52	518.85	0	303.68	1.71	0.00
Q8R081.2	Heterogeneous nuclear ribonucleoprotein L	63.97	1860.29	1008.37	1089.46	1.71	0.93
P63085.3	Mitogen-activated protein kinase 1	41.28	6838.61	7231.72	4005.4	1.71	1.81
Q8K310.1	Matrin-3	94.65	48934.68	44054.5	28726.06	1.70	1.53
P70333.1	Heterogeneous nuclear ribonucleoprotein H2	49.29	3343.55	3199.39	1967.64	1.70	1.63
Q60930.2	Outer mitochondrial membrane protein porin 2	31.74	86322.81	78898.69	50823.75	1.70	1.55
P62631.1	Elongation factor 1-alpha 2	50.46	29324.97	23360.65	17292.01	1.70	1.35
P48962.4	Adenine nucleotide translocator 1	32.91	21196.68	26120.94	12531.9	1.69	2.08
Q9CPQ1.3	Cytochrome c oxidase polypeptide Vic	8.47	26138.95	25535.01	15501.89	1.69	1.65
Q6IRU5.1	Clathrin light chain B	25.18	9545.47	5788.11	5698.24	1.68	1.02
P68372.1	Tubulin beta-2C chain	49.84	55580.54	58988.21	33302.34	1.67	1.77
P00493.3	Hypoxanthine-guanine phosphoribosyltransferase	24.57	5037.01	3204.2	3022.5	1.67	1.06
Q78ZA7.1	Nucleosome assembly protein 1-like 4	42.68	4688.42	0	2813.33	1.67	0.00
P97450.1	ATP synthase-coupling factor 6	12.5	9580.5	10919.41	5756.27	1.66	1.90
Q64433.2	Chaperonin 10	10.96	42888.72	36324.86	25830.45	1.66	1.41
O54774.1	Delta-adaptin	135.1	11197.1	8886.57	6781.64	1.65	1.31
P62823.1	Ras-related protein Rab-3C	25.88	24501.54	22919.68	14860.55	1.65	1.54
Q9CZC8.1	Secernin-1.	46.33	2759.81	5907.6	1674.48	1.65	3.53
Q8VDQ1.2	Prostaglandin reductase 2	38.02	2668.12	2954.5	1623.99	1.64	1.82
Q99LX0.1	Parkinson disease protein 7 homolog	20.02	27806.41	24268.5	16926.18	1.64	1.43
Q9D1L0.1	Coiled-coil-helix-coiled-coil-helix domain-containing protein 2	15.57	14434.87	12695.1	8793.22	1.64	1.44
O55022.4	Membrane-associated progesterone receptor component 1	21.7	11051.6	12019.41	6764.49	1.63	1.78
P26883.2	Immunophilin FKBP12	11.92	21108.04	22461.28	12930.49	1.63	1.74
O54828.2	Regulator of G-protein signaling 9	76.99	9064.08	9212.2	5554.58	1.63	1.66
P42669.1	Purine-rich single-stranded DNA-binding protein alpha	34.89	24853.18	30339.65	15255.83	1.63	1.99
Q3UEB3.2	Poly(U)-binding-splicing factor PUF60	60.26	7668.19	7520.47	4718.72	1.63	1.59
P99027.3	60S acidic ribosomal protein P2	11.65	87858.26	96569.94	54072.75	1.62	1.79
Q9R0P5.3	Destrin	18.52	16021.47	13597.53	9861.98	1.62	1.38
Q62188.1	Dihydropyrimidinase-related protein 3	34.13	177946.48	191194.91	109786.19	1.62	1.74
Q9DCN2.3	NADH-cytochrome b5 reductase 3	34.13	52600.13	58693.28	32584.27	1.61	1.80
P57722.3	Poly(rC)-binding protein 3	39.3	20374.21	22930.49	12671.46	1.61	1.81
O08583.3	Transcriptional coactivator Aly/REF	26.95	13729.9	11776.16	8552.98	1.61	1.38

P14211.1	Calreticulin	48	7535.68	7777.7	4707.23	1.60	1.65
Q8BVQ5.5	Protein phosphatase methylesterase 1	46.26	24915.76	23615.71	15587.46	1.60	1.52
Q8CGF7.2	Transcription elongation regulator 1	123.81	12884.63	14266.85	8066.45	1.60	1.77
Q9JKF6.2	Nectin-1	57.07	14195.45	11834.21	8895.24	1.60	1.33
P70168.1	Importin subunit beta-1	97.17	12313.79	4994.65	7720.5	1.59	0.65
P62317.1	Small nuclear ribonucleoprotein Sm D2	13.53	15219.46	17472.35	9553.65	1.59	1.83
Q8VDN2.1	Sodium pump subunit alpha-1	113	30068.42	24697.92	18882.26	1.59	1.31
P63017.1	Heat shock 70 kDa protein 8	70.88	14470.35	12534.41	9114.09	1.59	1.38
Q61474.1	RNA-binding protein Musashi homolog 1	39.12	2114.43	1358.98	1331.82	1.59	1.02
Q9DCJ5.3	NADH-ubiquinone oxidoreductase 19 kDa subunit	20	1523.44	2674.17	961.22	1.58	2.78
P27773.2	Protein disulfide-isomerase A3	56.68	6861.44	8210.18	4330.56	1.58	1.90
P62962.2	Profilin-1	14.96	3530.21	3465.59	2229.31	1.58	1.55
Q8BHN3.1	Alpha-glucosidase 2	106.93	1737.55	2790.24	1102.03	1.58	2.53
Q9CQQ7.1	ATPase subunit b	28.95	19804.66	18395.54	12581.66	1.57	1.46
P40142.1	Transketolase	67.64	77250.12	78678.14	49097.62	1.57	1.60
P29341.1	Polyadenylate-binding protein 1	70.66	11309.3	14966.56	7193.79	1.57	2.08
O35098.1	Dihydropyrimidinase-related protein 4	61.97	208125.05	196624.27	132442.27	1.57	1.48
O08709.3	Antioxidant protein 2	24.87	5246.89	4804.71	3349.32	1.57	1.43
P99024.1	Tubulin beta-5 chain	49.68	35732.88	31109.27	22835.09	1.56	1.36
P63101.1	Protein kinase C inhibitor protein 1	27.78	3925.91	5002.55	2509.35	1.56	1.99
Q8R326.1	Paraspeckle protein 1	58.77	15636.72	17555.86	9997.11	1.56	1.76
Q99104.1	Myosin-Va	215.64	2885.22	3132.02	1846.2	1.56	1.70
P60710.1	Beta-actin	41.74	14159.48	14878.57	9068.1	1.56	1.64
Q9JM14.1	5' 3'-deoxyribonucleotidase	23.08	10756.7	10583.3	6890.03	1.56	1.54
Q60737.1	Casein kinase II subunit alpha	45.17	48913.69	56470.19	31384.08	1.56	1.80
P31938.2	MAPK/ERK kinase 1	43.48	2660.71	4882.98	1709.81	1.56	2.86
P35979.2	60S ribosomal protein L12	17.81	9899.08	9495.12	6364.3	1.56	1.49
Q91WV0.1	TATA-binding protein-associated phosphoprotein	19.44	3866.73	645.47	2495.65	1.55	0.26
Q64471.3	Glutathione S-transferase theta-1	27.38	7325.74	4851.39	4736.34	1.55	1.02
Q91WN1.2	DnaJ homolog subfamily C member 9	30.07	9359.23	5213.03	6074.63	1.54	0.86
P12660.2	Purkinje cell protein 2	13.06	3854.11	3683.36	2503.09	1.54	1.47
Q8CG79.3	Tumor suppressor p53-binding protein 2	125.33	58566.52	64441.6	38069.02	1.54	1.69
P32020.3	Propanoyl-CoA C-acyltransferase	59.14	16362.79	16477.86	10640.04	1.54	1.55
Q99L47.1	Hsc70-interacting protein	41.66	18043.85	17094.99	11742.64	1.54	1.46
Q9Z2R6.1	Retinal protein 4	27.01	7393.17	10256.57	4820.84	1.53	2.13
Q9R1P3.1	Proteasome component C7-I	22.91	4910.59	4699.09	3203.38	1.53	1.47
Q91WK5.1	Glycine cleavage system H protein, mitochondrial	18.59	29152.96	27278.55	19080.8	1.53	1.43
P61082.1	Ubiquitin-conjugating enzyme E2	20.9	12344.41	12946.11	8094.97	1.52	1.60
P28798.2	Granulins	63.47	5032.19	3875.12	3302.48	1.52	1.17
Q922R8.3	Protein disulfide-isomerase A6	48.11	14746.98	12496.35	9686.21	1.52	1.29

P09411.4	Phosphoglycerate kinase 1	44.55	112991.93	90902.56	74424.99	1.52	1.22
P10852.1	4F2 cell-surface antigen heavy chain	58.35	3693.07	4069.42	2436.96	1.52	1.67
Q8JZS0.2	Protein lin-7 homolog A	26	3239.78	4791.91	2139.62	1.51	2.24
P61979.1	Heterogeneous nuclear ribonucleoprotein	50.98	11238.96	13009.58	7423.2	1.51	1.75
P50396.3	Guanosine diphosphate dissociation inhibitor 1	50.53	17940.1	22501.11	11851.35	1.51	1.90
P05201.2	Transaminase A	46.24	67528.32	62905.48	44731.49	1.51	1.41
P34022.2	GTPase-activating protein	23.6	3153.38	2792.21	2090.1	1.51	1.34
Q9Z1N5.1	Spliceosome RNA helicase Bat1	49.04	7257.72	9968.06	4811.44	1.51	2.07
P62897.2	Cytochrome c, somatic	11.61	8225.92	4164.29	5467.91	1.50	0.76
P60879.1	Synaptosomal-associated protein 25	23.32	3639.82	3829.28	2430.72	1.50	1.58
P18572.2	Basigin	42.45	5095.64	5813.1	3412.28	1.49	1.70
O88844.1	Cytosolic NADP-isocitrate dehydrogenase	46.67	62252.51	49546.74	41820.26	1.49	1.18
Q61792.1	Metastatic lymph node gene 50 protein	30	5081.66	1572.71	3415.22	1.49	0.46
Q9D819.1	Pyrophosphate phospho-hydrolase	32.67	29126.49	28322.49	19593.04	1.49	1.45
Q8VEK3.1	Heterogeneous nuclear ribonucleoprotein U	87.93	14392.91	16403.8	9690.95	1.49	1.69
P07901.4	Heat shock protein HSP 90-alpha	84.8	80664.94	99180.67	54433.51	1.48	1.82
Q9DBP5.1	Cytidylate kinase	22.17	8749.92	6077.56	5911.94	1.48	1.03
Q9CQA3.1	Succinate dehydrogenase [ubiquinone]	31.82	18010.69	15359.64	12206.96	1.48	1.26
Q921F2.1	TAR DNA-binding protein 43	44.56	8186.89	5153.05	5550.3	1.48	0.93
P56376.2	Acylphosphatase-1	11.24	7971.94	6495.26	5409.75	1.47	1.20
Q64105.1	Sepiapterin reductase	27.89	6546.8	6620.97	4458.83	1.47	1.48
Q62446.2	Rotamase	25.15	15403.87	16176.45	10503.48	1.47	1.54
P29595.2	Neural precursor cell expressed developmentally down-regulated gene 8	8.97	185547.49	207121.96	126530.28	1.47	1.64
P01831.1	Thymus cell antigen 1, theta	18.08	27970.82	27413.01	19123.86	1.46	1.43
P19783.2	Cytochrome c oxidase subunit IV isoform 1	19.53	15764.39	21157.83	10779.69	1.46	1.96
P97822.2	Cerebellar postnatal development protein 1	29.63	6064.33	6096.63	4165.62	1.46	1.46
Q9D051.1	Pyruvate dehydrogenase (lipoamide) beta	38.94	11468.49	17632.54	7884.04	1.45	2.24
P63011.1	RAB3A, member RAS oncogene family	24.97	5337.77	5122.07	3678.3	1.45	1.39
P17182.3	Enolase 1, alpha non-neuron	47.15	89479.63	99464.56	61722.92	1.45	1.61
Q93092.2	Transaldolase	37.39	3745.81	3236.11	2584.22	1.45	1.25
Q9CR51.3	Vacuolar proton pump subunit G 1	13.73	5820.25	4087.36	4022.92	1.45	1.02
P62874.3	Guanine nucleotide binding protein (G protein), beta 1	37.38	9712.35	7565.22	6728.06	1.44	1.12
P35235.2	Tyrosine-protein phosphatase non-receptor type 11	68.47	1138.56	1393.79	789.24	1.44	1.77
P06745.4	Glucose phosphate isomerase 1	62.78	33157.72	34937.79	23038.51	1.44	1.52
Q9D6Y7.1	Peptide methionine sulfoxide reductase	25.99	58098.3	53104.66	40378.19	1.44	1.32
P16125.2	L-lactate dehydrogenase B chain	36.58	26982.93	28990.51	18763.51	1.44	1.55

O88569.2	Heterogeneous nuclear ribonucleoproteins A2/B1	37.41	4112.23	4152.28	2865.65	1.44	1.45
P62821.3	Ras-related protein Rab-1A	22.68	4857.09	1138.5	3393.94	1.43	0.34
P63038.1	60 kDa heat shock protein, mitochondrial	60.96	17767	15875.03	12421.1	1.43	1.28
P09103.1	Protein disulfide-isomerase	57.15	7972.94	5698.07	5587.37	1.43	1.02
P22599.2	Alpha-1-antitrypsin 1-2	45.98	2510.97	3139.86	1769.48	1.42	1.77
P45376.3	Aldose reductase	35.74	6958.63	7688.68	4905.42	1.42	1.57
O54751.1	Cone-rod homeobox protein	32.38	3951.83	1532.88	2793.16	1.41	0.55
Q9D0S9.1	Histidine triad nucleotide-binding protein-2	17.32	26859.14	22941.72	19006.88	1.41	1.21
Q62348.1	Translin	26.2	26684.04	37188.12	18964.83	1.41	1.96
P10639.3	Thioredoxin	11.68	6387.62	5411.65	4545.48	1.41	1.19
Q9WVL4.1	Rhodopsin kinase	63.58	36829.11	52058.2	26252.03	1.40	1.98
O08553.2	Dihydropyrimidinase-related protein 2	62.29	300713.55	373455.2	214406.42	1.40	1.74
P30275.1	Acidic-type mitochondrial creatine kinase	47.01	23961.89	24792.58	17087.74	1.40	1.45
P14206.4	Laminin-binding protein precursor p40	32.84	7821.25	4091.14	5603.11	1.40	0.73
P14733.3	Lamin-B1	66.8	1013.6	1261.76	727.42	1.39	1.73
Q60597.3	Alpha-ketoglutarate dehydrogenase	116.47	10419.3	12397.87	7533.24	1.38	1.65
Q9QYG0.1	Protein Ndr2	40.8	33179.49	31349.76	23990.2	1.38	1.31
P51863.2	Vacuolar proton pump subunit d 1	40.31	3703.66	3037.91	2691.83	1.38	1.13
P17183.2	Enolase 2, gamma neuronal	47.3	37228.59	41634.02	27184.15	1.37	1.53
Q3URD3.2	Sarcolemmal membrane-associated protein	96.96	7620.01	6381.27	5586.07	1.36	1.14
Q3UM45.2	Protein phosphatase 1 regulatory subunit 7	41.3	8161.75	10169.92	6017.19	1.36	1.69
Q9CQ54.1	NADH-ubiquinone oxidoreductase subunit B14.5b	14.17	3354.09	2670.5	2484.43	1.35	1.07
Q8BFR5.1	Tu translation elongation factor, mitochondrial	49.51	41728.22	42180.87	31015.22	1.35	1.36
Q60605.3	Myosin light polypeptide 6	16.93	1907.91	2661.39	1418.76	1.34	1.88
Q03265.1	ATP synthase subunit alpha, mitochondrial	59.76	16986.85	16121.47	12678.98	1.34	1.27
Q9Z2I9.2	Succinyl-CoA synthetase beta-A chain	50.12	14652.46	11873.55	10987.46	1.33	1.08
P09671.3	Superoxide dismutase [Mn], mitochondrial	24.61	2135.53	3495.57	1606.77	1.33	2.18
Q99KJ8.3	Dynactin subunit 2	44.13	2744.04	2567.86	2064.91	1.33	1.24
P12970.2	60S ribosomal protein L7a	29.98	10759.2	8227.3	8110.56	1.33	1.01
P11983.3	T-complex protein 1	60.64	16726.9	16281.2	12609.89	1.33	1.29
P47955.1	60S acidic ribosomal protein P1	11.48	5259.23	2601.44	3972.84	1.32	0.65
P19157.2	Glutathione S-transferase, pi 1	23.61	12938.27	10921.71	9949.3	1.30	1.10
P05064.2	Fructose-bisphosphate aldolase A	39.36	130142.05	143286.62	100401.4	1.30	1.43
Q64520.2	Guanylate kinase	21.92	33841.01	29421.94	26155.73	1.29	1.12
Q62425.2	NADH-ubiquinone oxidoreductase MLRQ subunit	9.33	18320.52	22522.85	14217.38	1.29	1.58
Q9D1A2.1	Cytosolic non-specific dipeptidase	52.77	7748.54	7261.38	6019.41	1.29	1.21
P15105.6	Glutamate-ammonia ligase	42.13	28248.53	36378.68	21964.27	1.29	1.66
Q9D0E1.3	Heterogeneous nuclear	77.66	1808.8	1457.77	1409.23	1.28	1.03

	ribonucleoprotein M						
P68369.1	Tubulin alpha-1A chain	50.14	15041.45	15294.12	11753.3	1.28	1.30
P99029.2	Peroxiredoxin 5	21.9	7299.97	9972.18	5712.35	1.28	1.75
Q8BYK4.1	Retinol dehydrogenase 12	35.3	13197.55	11735.13	10376.82	1.27	1.13
Q571F8.2	Glutaminase liver isoform, mitochondrial	66.38	8573.18	12056.2	6789.99	1.26	1.78
O35887.1	Calumenin	37.07	5498.17	2191.33	4402.12	1.25	0.50
P07146.1	Anionic trypsin-2	26.21	78395.48	90151.73	62846.88	1.25	1.43
Q8BMF4.2	Pyruvate dehydrogenase complex component E2	67.95	5880.73	9780.67	4716.56	1.25	2.07
Q91X78.1	Endoplasmic reticulum lipid raft-associated protein 1	38.94	18463.32	17382.82	14889.94	1.24	1.17
Q61171.3	Peroxiredoxin 2	21.78	10190.91	17519.27	8254.84	1.23	2.12
Q11011.2	Puromycin-sensitive aminopeptidase	103.34	11016.61	12161.7	8923.92	1.23	1.36
P27664.2	Aminopeptidase puromycin sensitive	103.34	13383	14510.94	10842.32	1.23	1.34
Q8BG39.1	Synaptic vesicle glycoprotein 2B	77.47	14708.68	15834.34	11922.58	1.23	1.33
O35737.3	Heterogeneous nuclear ribonucleoprotein H	49.21	2310.32	3351.9	1889.58	1.22	1.77
Q62420.2	Endophilin-A1	39.96	2207.99	3438.56	1808.09	1.22	1.90
P45952.1	Medium-chain specific acyl-CoA dehydrogenase, mitochondrial	46.49	7626.89	4850.89	6273.68	1.22	0.77
Q60714.1	Long-chain fatty acid transport protein 1	71.29	4208.7	4568.6	3489.35	1.21	1.31
P00920.4	Carbonic anhydrase 2	29.04	11325.84	18471.31	9392.62	1.21	1.97
Q61696.2	Heat shock protein 1A	70.09	1972.17	0	1636.83	1.20	0.00
Q9D0F9.3	Phosphoglucomutase-1	61.53	6687.21	5064.92	5556.76	1.20	0.91
Q91VD9.1	NADH-ubiquinone oxidoreductase 75 kDa subunit, mitochondrial	79.76	6417.38	7355.6	5380.8	1.19	1.37
Q9D6R2.1	Isocitric dehydrogenase subunit alpha	39.64	33801.36	28495.38	28364.91	1.19	1.00
Q99K48.3	Non-POU domain-containing octamer-binding protein	54.56	3841.4	4379.54	3233.17	1.19	1.35
Q9CPU0.3	Lactoylglutathione lyase	20.81	9702.19	8809.41	8182.49	1.19	1.08
Q9CQM5.1	Thioredoxin domain-containing protein 17	14.02	9535.02	7485.09	8054.14	1.18	0.93
Q80WM3.2	Complexin 4	18.36	43171.76	47546.18	36523.2	1.18	1.30
Q08331.3	Calretinin	31.38	11400.82	13544.94	9655.38	1.18	1.40
Q9CWS0.3	Dimethylarginine dimethylaminohydrolase 1	31.39	19661.55	31054.02	16734.91	1.17	1.86
P05202.1	Aspartate aminotransferase, mitochondrial	47.42	16022.53	25993.13	13714.14	1.17	1.90
P51881.3	Adenine nucleotide translocator 2	32.94	13039.84	18347.63	11244.75	1.16	1.63
P17182.3	Alpha-enolase	47.15	30401.58	37549.48	26401.05	1.15	1.42
Q63844.5	Mitogen-activated protein kinase 3	43.07	3787.21	5974.11	3290.31	1.15	1.82
P08249.3	Malate dehydrogenase, mitochondrial	35.62	39858.12	54041.01	34632.58	1.15	1.56
Q91VM9.1	Inorganic pyrophosphatase 2, mitochondrial	38.12	5656.03	8371.48	4950.29	1.14	1.69
Q61017.2	Guanine nucleotide-binding protein G(I)/G(S)/G(O) subunit gamma-T2	7.8	7868.12	9830.85	6962.25	1.13	1.41
P62881.1	Guanine nucleotide-binding protein subunit beta-5	43.57	3960.64	3772.86	3533.84	1.12	1.07
Q8VEM8.1	Phosphate transport protein	39.64	12821.19	14855.94	11473.91	1.12	1.29

Q9D2M8.4	Ubiquitin-conjugating enzyme E2 variant 2	16.37	13706.92	12773.49	12273.67	1.12	1.04
Q9CR16.3	Peptidyl-prolyl cis-trans isomerase	40.75	11367.57	14524.91	10278.29	1.11	1.41
Q9CPV4.1	Glyoxalase domain-containing protein 4	33.32	26094.13	25860.82	23895.45	1.09	1.08
P14824.2	Annexin A6	75.9	17868.48	24073.34	16448.4	1.09	1.46
Q8K2B3.1	Succinate dehydrogenase [ubiquinone] flavoprotein subunit, mitochondrial	72.6	4771.37	4594.93	4393.63	1.09	1.05
Q9DCX2.3	ATP synthase subunit d, mitochondrial	18.75	1510.36	2670.28	1399	1.08	1.91
Q9D0M5.1	Dynein light chain LC8-type 2	10.35	6620.99	12958.48	6204.44	1.07	2.09
P56391.2	Cytochrome c oxidase subunit 6B1	10.07	9972.81	15618.63	9357.85	1.07	1.67
P32958.1	Rod outer segment membrane protein 1	37.27	22465.26	30953.78	21106.48	1.06	1.47
P05063.4	Fructose-bisphosphate aldolase C	39.4	6499.43	9860.58	6124.04	1.06	1.61
Q9D3D9.1	ATP synthase subunit delta, mitochondrial	17.6	7337.19	5390.94	6920.98	1.06	0.78
P97807.2	Fumarate hydratase, mitochondrial	54.38	1403.72	1709.05	1327.29	1.06	1.29
P34057.2	Rocoverin	23.41	6000.89	3383.9	5698.58	1.05	0.59
Q9JII6.3	Aldehyde reductase	36.59	5173.92	4216.2	4922.57	1.05	0.86
B2RUR4.1	Keratinocyte proline-rich protein	71.76	4134.9	4697.1	3942.26	1.05	1.19
P20443.1	Retinal S-antigen	44.94	6716.54	7305.05	6408.57	1.05	1.14
Q61548.1	Clathrin coat assembly protein AP180	91.86	1109.56	1831.02	1086.51	1.02	1.69
Q9CZU6.1	Citrate synthase, mitochondrial	51.74	1706.97	2583.13	1698.95	1.00	1.52
Q9DCD0.3	6-phosphogluconate dehydrogenase	53.26	2557.77	2393.5	2555.71	1.00	0.94
Q8K4Z3.1	Apolipoprotein A-I-binding protein	30.98	5764.64	6719.28	5803.97	0.99	1.16
P15409.1	Rhodopsin	39.03	3841.49	1350.52	3880.86	0.99	0.35
P05213.2	Tubulin alpha-1B chain	50.16	3509.66	5144.37	3549.35	0.99	1.45
Q9CQF3.1	Cleavage and polyadenylation specificity factor subunit 5	26.25	3500.06	3366.52	3542.51	0.99	0.95
P48758.3	NADPH-dependent carbonyl reductase 1	30.65	6581.27	8582.09	6710.76	0.98	1.28
P48036.1	Annexin A5	35.76	6910.18	12702.26	7061.86	0.98	1.80
Q9DCW4.3	Electron transfer flavoprotein subunit beta	27.63	23899.34	21540.07	24434.28	0.98	0.88
Q60597.3	2-oxoglutarate dehydrogenase complex<br>component E1	116.47	4333.8	2059.44	4451.95	0.97	0.46
Q00612.3	Glucose-6-phosphate 1-dehydrogenase X	59.27	1232.65	1333.69	1267.91	0.97	1.05
P68368.1	Tubulin alpha-4A chain	49.93	6161.89	6373.54	6344.18	0.97	1.00
Q9JKD3.1	Secretory carrier-associated membrane protein 5	26.07	7445.48	14437.68	7743.27	0.96	1.86
P62075.1	Mitochondrial import inner membrane translocase subunit Tim13	10.46	1843.96	2322.47	1963.04	0.94	1.18
Q91X72.1	Hemopexin	51.35	2569.45	3519.17	2745.19	0.94	1.28
P17751.3	Triosephosphate isomerase	26.72	981.78	1724.97	1073.81	0.91	1.61
P09405.2	Nucleolin	76.73	4400.33	9552.07	4848.27	0.91	1.97
P52480.4	Pyruvate kinase muscle isozyme M1/M2	57.85	6007.47	12319.36	6886.53	0.87	1.79
P61922.1	4-aminobutyrate aminotransferase,	56.46	8809.67	6029	10181.14	0.87	0.59

	mitochondrial						
Q61598.1	Guanosine diphosphate dissociation inhibitor 2	50.54	1304.01	985.2	1512.37	0.86	0.65
P49194.3	Retinol-binding protein 3	134.47	35269.07	22790.55	40972.94	0.86	0.56
P67778.1	Prohibitin	29.83	2679.38	2747.24	3130.95	0.86	0.88
Q9CZ44.1	p97 cofactor p47	40.72	1029.14	0	1268.66	0.81	0.00
P09174.1	Retinal rod rhodopsin-sensitive cGMP 3',5'-cyclic phosphodiesterase subunit gamma	9.64	7662.38	9130.51	9575.94	0.80	0.95
P52503.2	NADH-ubiquinone oxidoreductase 13 kDa-A subunit	13.02	869.96	633.3	1094.25	0.80	0.58
P62814.1	Vacuolar proton pump subunit B 2	56.56	2339.58	2572.93	2953.1	0.79	0.87
Q9EP71.1	Retinoic acid-induced protein 14	108.08	3653.89	5338.87	4686.06	0.78	1.14
P27546.3	Microtubule-associated protein 4	117.45	855.09	383.34	1117.41	0.77	0.34
P17809.3	Glucose transporter type 1, erythrocyte/brain	53.94	3684.66	9443.52	4837.93	0.76	1.95
P15499.1	Retinal degeneration slow protein	39.26	8905.9	15421.94	11879.78	0.75	1.30
Q99KI0.1	Aconitase hydratase, mitochondrial	85.48	1993.78	1867.84	2681.46	0.74	0.70
Q921I1.1	Serotransferrin	76.73	2642.53	4720.96	3560.57	0.74	1.33
Q9D3A9.1	Protein tweety homolog 1	49.04	2511.88	2466.45	3388.76	0.74	0.73
O88342.3	Actin-interacting protein 1	66.41	4787.73	7575.24	6643.07	0.72	1.14
P27546.3	Microtubule-associated protein 4	117.45	275.52	598.97	402.33	0.68	1.49
Q9CPQ8.1	ATP synthase subunit g, mitochondrial	11.43	2082.21	4385.03	3053.01	0.68	1.44
O08976.2	Probasin	20.38	26372.59	32807.65	39200.98	0.67	0.84
P06801.1	NADP-dependent malic enzyme	64.01	1084.34	2229.28	1612.96	0.67	1.38
O08599.2	Syntaxin-binding protein 1	67.58	226.26	757.66	378.09	0.60	2.00
P04247.3	Myoglobin	17.07	2098.5	781.91	3514.57	0.60	0.22
Q8C0M9.1	L-asparaginase	33.95	871.68	2270	1476.76	0.59	1.54
Q8K0T0.1	Reticulon-1	83.59	380.69	677.67	680.44	0.56	1.00
O08585.1	Clathrin light chain A	25.56	2569.7	4565.74	4737.19	0.54	0.96
P57746.1	Vacuolar proton pump subunit D	28.37	442.02	1459.57	823.26	0.54	1.77
P50247.3	Adenosylhomocysteinase	47.69	2025.84	4219.91	3889.91	0.52	1.08
O08976.2	Probasin	20.38	7554.27	6566.12	14589.21	0.52	0.45
P02815.1	Salivary protein 1	14.9	12680.64	5718.15	24586.55	0.52	0.23
Q6PDM2.3	Splicing factor, arginine/serine-rich 1	27.75	541.26	942.15	1061.18	0.51	0.89
Q9D2G2.1	2-oxoglutarate dehydrogenase complex component E2	49	394.27	0	813.02	0.48	0.00
Q8QZT1.1	Acetoacetyl-CoA thiolase	44.82	1703.96	4261.44	3817.38	0.45	1.12
P62192.1	26S protease regulatory subunit 4	49.19	277	1652.09	651.05	0.43	2.54
Q9WV34.1	MAGUK p55 subfamily member 2	61.57	202.95	531.72	525.45	0.39	1.01
O08749.2	Dihydrolipoamide dehydrogenase	54.28	700.22	0	1860.22	0.38	0.00
O70318.1	Band 4.1-like protein 2	109.85	178.93	324.47	484.9	0.37	0.67
Q9D517.2	Lysophosphatidic acid acyltransferase gamma	43.3	287.28	1338.05	869.17	0.33	1.54
P10649.2	Glutathione S-transferase Mu 1	25.97	1729.98	2842.55	5662.55	0.31	0.50
P07310.1	Creatine kinase M-type	43.05	11340.89	10513.62	37826.51	0.30	0.28

P47754.3	F-actin-capping protein subunit alpha-2	32.97	499.91	1174.93	2244.32	0.22	0.52
O70250.3	Phosphoglycerate mutase 2	28.83	2478.61	5030.17	12911.49	0.19	0.39
Q8R429.1	Sarcoplasmic/endoplasmic reticulum calcium ATPase 1	109.44	1248.09	2362.68	6851.04	0.18	0.34
P32848.3	Parvalbumin alpha	11.93	2042.29	3104.07	11694.83	0.17	0.27
P21550.3	Skeletal muscle enolase	47.03	498.13	1332.36	4512.41	0.11	0.30
Q9Z1G4.2	Vacuolar proton pump subunit 1	96.52	0	667.56	723.82	0.00	0.92
Q91XV3.3	Neuronal axonal membrane protein NAP-22	22.09	0	0	338.21	0.00	0.00
Q9D6Y9.1	Glycogen-branching enzyme	80.37	0	299.48	409.97	0.00	0.73
O54984.2	Arsenite-stimulated ATPase	38.83	0	1533.96	939.77	0.00	1.63
P16858.2	Glyceraldehyde-3-phosphate dehydrogenase	35.81	0	0	370.38	0.00	0.00
P84091.1	Mu2-adaptin	49.66	0	0	315.11	0.00	0.00
P70296.3 Q8VIN1.1	Phosphatidylethanolamine binding protein 1	20.83	0	0	0	Undef	Undef
Q61937.1	Nucleophosmin 1	32.57	0	0	0	Undef	Undef
Q9ERD7.1	Tubulin beta-3 chain	50.43	0	0	0	Undef	Undef
Q9WV92.1	Band 4.1-like protein	103.36	0	0	0	Undef	Undef
Q64213.5	Splicing factor 1	70.42	0	731.52	0	Undef	Undef
Q8BK64.2	Activator of 90 kDa heat shock protein ATPase homolog 1	38.12	0	0	0	Undef	Undef
Q8CIN4.1 O88643.1	Serine/threonine-protein kinase PAK 2	57.94	0	0	0	Undef	Undef
O70443.4	Guanine nucleotide binding protein, alpha z subunit	40.86	0	3016.61	0	Undef	Undef
Q9Z1L4.1	Retinoschisin	25.58	0	2769.02	0	Undef	Undef
O08547.3	Vesicle-trafficking protein SEC22b	24.75	0	0	0	Undef	Undef
Q921U8.2	Smoothelin	100.31	0	396.43	0	Undef	Undef

Ratios were determined by dividing total area of the designated experimental group by total area of the control group

*Undef: undefined

Table S2. PCR primers. Commercially designed (Applied Biosystems)

Gene Symbol	Assay ID number
Ccl2	Mm00441242_m1
Nos2	Mm00440502_m1
Il1b	Mm00434228_m1
Tnfa	Mm00443258_m1
Cxcl10	Mm00445235_m1
Arg1	Mm00475988_m1
Cd163	Mm00474091_m1
Mrc1	Mm00485148_m1
Gapdh	Mm99999915_g1
